# Modelling *Aedes albopictus* management, incorporating immigration and bi-directional *Wolbachia* interactions

**DOI:** 10.1007/s10340-026-02030-4

**Published:** 2026-03-10

**Authors:** Matthew Ryan, Manuela Mendiolar, Dan Pagendam, Roslyn I. Hickson, Brendan Trewin

**Affiliations:** 1 Commonwealth Scientific and Industrial Research Organisation (CSIRO), Adelaide, Australia; 2 Commonwealth Scientific and Industrial Research Organisation (CSIRO), Brisbane, Australia; 3 Australian Institute for Tropical Health and Medicine, James Cook University, Townsville, Australia; 4 Commonwealth Scientific and Industrial Research Organisation (CSIRO), Townsville, Australia

**Keywords:** Vector control, Incompatible insect technique, Suppression, Bi-directional cytoplasmic incompatibility, Reversibility

## Abstract

*Aedes albopictus* mosquitoes are competent vectors for the spread of at least 24 different arboviruses, including dengue, Ross River, and Japanese encephalitis viruses. However, they remain less studied than their more urban cousins, *Aedes aegypti*. We model an incompatible insect technique (IIT) strategy for mosquito control, with bi-directional incompatibility between two strains of *Wolbachia* (*w*AlbA/*w*AlbB  $\times$ AR*w*P) and age-based cytoplasmic incompatibility decay in a well-mixed population. We include mosquito immigration to explore potential reversibility, an important consideration in bi-directional IIT control programs. We also explore the establishment probability after female contamination of an artificially-infected *Wolbachia* mosquito strain, consider the suppression dynamics and probability of mosquito management success for different release strategies, and determine a corresponding cost proxy for release (numbers of mosquitoes released). We found an establishment probability threshold of 40% in the absence of mating preferences, though this threshold needs validation in future field and laboratory experiments. We found differences in suppression success between release cessation and 6 months later for different immigration rates. There are similar short-term costs with differences in medium- and longer-term costs between release strategies. Our model suggests bi-directional IIT control programs are reversible with low amounts of wild-type immigration. This work demonstrates opportunities to optimise the suppression of these medically important mosquitoes.

**Supplementary Information:**

The online version contains supplementary material available at 10.1007/s10340-026-02030-4.

## Introduction

*Aedes albopictus* is the most invasive mosquito species in the world (2024), (Benedict et al. 2007). This species is capable of transmitting many pathogens to humans including chikungunya, Zika, and Japanese encephalitis (Zhang et al. 2023; Leta et al. 2018) and is responsible for spreading dengue into areas where the disease had once been eliminated (Cochet et al. 2022; Quam et al. 2016; Zeng et al. 2022). Most control programs for *Ae. albopictus* rely on traditional approaches, including removal of breeding sites in urban areas and the application of chemical insecticides (Fonseca et al. 2013). Resistance to these chemical interventions is increasing (Auteri et al. 2018; Asgarian et al. 2023), while removal of larval habitat is labour-intensive and inefficient, as it does not remove cryptic or inaccessible sites (Kay et al. 2000). As such, new and innovative population control strategies that scale across large urban environments are urgently needed.

*Wolbachia*-based population suppression and replacement strategies are well-established for the control of *Ae. aegypti* mosquitoes and the diseases they spread (see, for example, (Crawford et al. 2020; Zheng et al. 2019; Hoffmann et al. 2011; Nazni et al. 2019; Beebe et al. 2021). Cytoplasmic incompatibility (CI), a biological trait of the endosymbiotic *bacteria* *Wolbachia*, is an important component of both suppression (Beebe et al. 2021; Crawford et al. 2020; Zheng et al. 2019) and replacement control strategies (Hoffmann et al. 2011). This natural mechanism of reproductive manipulation by *Wolbachia* on its insect host phenotype is referred to as a uni-directional (one-way) CI and alters the outcomes of mating. Only crosses between both *Wolbachia*-infected male and female mosquitoes, or uninfected wild-type males and *Wolbachia*-infected females, produce viable offspring in the next generation (Barr 1980; Laven 1967). The incompatible insect technique (IIT) is a management technique which utilises CI, taking advantage of the uni-directional incompatibility exhibited by *Wolbachia*-infected males and a wild population that is uninfected (Boller and Bush 1974; Boller et al. 1976). Research trialling *Wolbachia* as a control strategy for *Ae. aegypti* have recently achieved successful suppression of populations in field trials (Crawford et al. 2020; Zheng et al. 2019; Beebe et al. 2021). While the *Ae. aegypti* uni-directional CI system is well studied, suppressing populations of other medically important mosquito species requires further investigation.

There has been substantially less focus on the control of *Ae. albopictus* via the IIT. *Ae. albopictus* exhibits different fitness characteristics when compared to *Ae. aegypti*, such as reproduction rate, average life expectancy, and an ability to overwinter in colder regions. Furthermore, cage experiments and modelling of *Ae. aegypti* systems to identify uni-directional invasion thresholds for *Wolbachia* establishment (see, for example, (Hoffmann et al. 2011; Pagendam et al. 2020)) are not directly applicable to *Ae. albopictus* as wild populations of the species carry *w*AlbA/*w*AlbB (henceforth abbreviated to *w*AlbAB unless otherwise specified) strains of *Wolbachia* and exhibit a natural form of CI(Ogunlade et al. 2022).

In recent years, a number of artificial *Wolbachia* infections have been introduced into *Ae. albopictus*, including single (Moretti and Calvitti 2013) and triple infections of the Pipientis *Wolbachia* (*w*Pip) from *Culex* mosquitoes (Zhang et al. 2015). In particular, the injection of *Ae. albopictus* with the AR*w*P form of *w*Pip has been successfully deployed in IIT field trials (Caputo et al. 2020; Mains et al. 2016). When two incompatible *Wolbachia*-infected mosquitoes mate, all subsequent offspring of these matings do not survive. This process is referred to as bi-directional CI (Moretti et al. 2018). Control programs applying a bi-directional CI strategy introduce several unknown factors impacting management decision-making, including the level of population suppression that can be achieved as influenced by *Wolbachia* invasion thresholds, particularly if *Wolbachia*-infected females are released through imperfect sex separation (Pagendam et al. 2020; Lombardi et al. 2024).

Despite increasing improvements to mosquito sex-separation technologies (Crawford et al. 2020), some methods are imperfect and have the potential to release artificially-infected *Wolbachia* females into the landscape, risking the undesired outcome of population replacement (Consortium and Ching 2021; Ross 2021). Typical sex-separation technologies using sieves result in a small female contamination rate of around $1-5$% (Consortium and Ching 2021). Establishing *Wolbachia* in an *Ae. aegypti*, IIT control program at a threshold of greater than $20\%$ should lead to a population replacement outcome (Zhang et al. 2015; Consortium and Ching 2021), as wild populations have historically not contained *Wolbachia*. In this case, population replacement is an undesirable outcome of an IIT control program as the released *Wolbachia* would no longer be effective at achieving its goal of suppression (Dobson et al. 2002).

Typically, a combination of both IIT and a radiation treatment (Sterile Insect Technique) have been used as a method to prevent unwanted establishment of an artificial *Wolbachia* strain (Soh et al. 2022; Zheng et al. 2019). However, natural *Ae. albopictus* populations containing *w*AlbAB exhibit CI and could theoretically suppress the establishment of an artificial *Wolbachia* strain, should females be released (Moretti et al. 2018). This opens the potential for management decisions which prevent establishment and continue suppression of the wild population with imperfect sex separation.

While establishing an artificial *Wolbachia* strain in a wild *w*AlbAB population may be minimised, this is not guaranteed, as the *Wolbachia* concentrations in wild *Ae. albopictus* males can decrease as they age. This can reduce the effectiveness of CI in *Ae. albopictus* from 100% at $1-15$ days to 68% at $15-19$ days, and 0% (i.e. no incompatibility) at 20 days and beyond (Calvitti et al. 2015). This decrease imparts a relative reproductive advantage to the AR*w*P *Ae. albopictus* population used in the IIT control program, as AR*w*P infected females can successfully mate with older wild-type males, while AR*w*P mosquitoes also exhibit marginally improved fitness to the wild *w*AlbAB population (Calvitti et al. 2015).

Thus, investigating IIT control programs for the management of *Ae. albopictus* presents interesting and unique challenges. The combination of different fitness parameters, bi-directional CI, and age-related decay in CI means that previous studies for *Ae. aegypti* are not immediately applicable to *Ae. albopictus* (Doeurk et al. 2025; Maimusa et al. 2016; Brady et al. n.d.). Further, a challenge for the regulation of these types of new technologies is the risk an IIT control program leads to unintended consequences, such as a population replacement rather than suppression. Given imperfect sex separation is common, there is a genuine concern of establishing an artificial *Wolbachia* stain when applying the IIT. This risk may be mitigated by natural immigration and emigration of wild *Ae. albopictus* populations from surrounding areas. To investigate the complex nature of a bi-directional IIT control program for *Ae. albopictus* while considering the impacts of immigration, we use mathematical modelling as a cost-effective tool to explore a range of population suppression scenarios.

Mathematical and computational models have been used to explore the impacts of multiple *Wolbachia* strains as a control agent for mosquito populations. These models typically focus on either understanding conditions of co-existence of multiple *Wolbachia* strains in the wild (Keeling et al. 2003; Ogunlade et al. 2022; Farkas and Hinow 2010; Telschow et al. 2005) or the effects of bi-directional CI on IIT control programs (Dobson et al. 2002; Moretti et al. 2018; Turelli 2010; Soh et al. 2022). In particular, Moretti et al. (Moretti et al. 2018) built upon the discrete generation model of Dobson et al. (Dobson et al. 2002) to understand how the introduction of AR*w*P *Wolbachia* may be used to control wild-type *Ae. albopictus* populations. In their approach, Moretti et al. used a deterministic model to investigate non-overlapping generations of mosquitoes. However, deterministic models can have significant limitations for small population sizes where small stochastic changes can have a large impact on the dynamics (Pagendam et al. 2020), and discrete generations precludes the modelling of the age-based CI decay found in wild-type *Ae. albopictus* mentioned above.

Here, we build on an existing stochastic computational model for *Ae. aegypti* developed by Pagendam et al. (2020) to explore suppression scenarios applying bi-directional CI and AR*w*P *Wolbachia* establishment thresholds on a theoretical population of *Ae. albopictus*. Using the extended model, we focus on hypothetical scenarios where AR*w*P-infected *Ae. albopictus* are introduced into a background of wild-type *w*AlbAB. We explore scenarios where populations of wild *Ae. albopictus* are managed without uncontrolled replacement by an established AR*w*P population and we determine how key differences in suppression affect management success, including a proxy for cost.

## Materials and methods

When constructing our model, we are motivated by modelling a single urban block of houses surrounded by many un-modelled patches (Manica et al. 2016; Pagendam et al. 2020). From a mathematical perspective, this means we restrict our investigations to a single-patch model. Our model can be extended to patches larger than an urban block (for example, an isolated island) by modifying the parameter estimates and initial conditions of the model. A description of all model parameters and their estimates used are found in Supplementary Table S1, with the stoichiometries fully described in Table S2.

### Model description

**Fig. 1:**
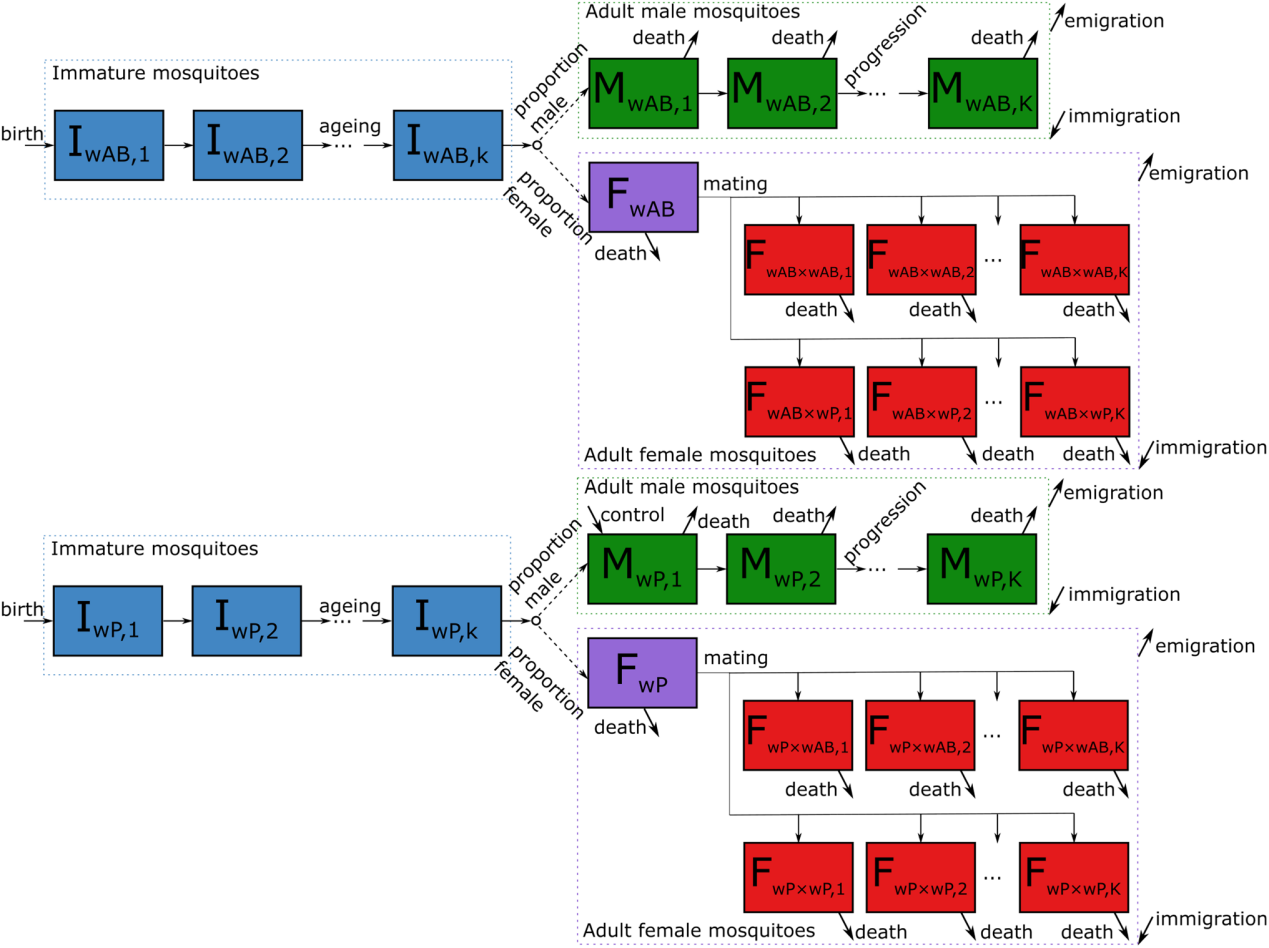
Schematic of the mosquito population model, showing progression from the immature stages (blue) through to adult males (green) or females who are unmated (purple). Females then progress to those mated with males with a specific *Wolbachia* type and Cytoplasmic Incompatibility efficacy (red). Immigration and emigration occurs from each adult compartment, captured here by the flows from the dashed boxes grouping compartments, though in the scenarios we study there is no immigration of AR*w*P mosquitoes. For readability, *w*AlbAB has been abbreviated to wAB and AR*w*P has been abbreviated as wP. In the $F_{wX \times wJ}$ notation the first strain (*wX*) refers to the *Wolbachia* strain the female carries, and the second (*wJ*) to the male

Pagendam et al. (2020) proposed a *Wolbachia*-IIT Markov Population Process model to investigate the efficacy of different release strategies on the risk of *Wolbachia* establishment for *Ae. aegypti* populations. We extend their model to *Ae. albopictus* and include age-related decay in *Wolbachia* CI, as well as immigration and emigration for wild adult mosquito populations (Fig. 1). Following Pagendam *et al.*, we assume constant fitness parameters (Table S1) and do not model seasonality or overwintering. Many components of our model correspond to the Pagendam et al. (2020) model, which we recap briefly for completeness.

Pagendam et al.’s model proposes that there is a pool of future adult mosquitoes, or “immature” mosquitoes, that spend a Gamma-distributed amount of time as immatures. Immature mosquitoes include the eggs, larvae, and pupae in the mosquito population that will survive to adulthood. When immatures age to become adults, there is a 50% chance that they will either become adult males or unmated adult females. Adult males can then mate with unmated females, and we assume that females only mate with one male in their lifespan. Male and female mosquitoes mate at a species-dependent rate, modified by Fried’s index. That is, if there are more male mosquitoes of a particular type (*w*AlbAB or AR*w*P), then females are more likely to mate with that type of male. Mated female adults then give birth to immatures at a rate $\tilde{\lambda }$. The birth rate of immatures is modified by a density-dependent rate that controls for the size of the immature pool; this emulates the density dependence of higher larval mortality at higher larval population densities. Note that both *w*AlbAB and AR*w*P immature mosquitoes contribute to the same immature pool and hence the density-dependent rate.

Our proposed model considers a bi-directional CI approach where mated females $F_{\textit{w}AlbAB\times AR\textit{w}P}$ and $F_{AR\textit{w}P\times \textit{w}AlbAB}$ fail to produce viable offspring, where the first subscript denotes the *Wolbachia* strain of the female mosquito, and the second subscript indicates the strain of the male. However, CI decays with the age of male *w*AlbAB mosquitoes, meaning that as male *w*AlbAB mosquitoes age there is a higher chance for $F_{AR\textit{w}P\times \textit{w}AlbAB}$ to produce viable AR*w*P offspring. To capture this age-related decay of CI, we discretise the male mosquitoes into *K* compartments, depicted in Fig. 1, where $K=20$ in our study. We use the CI values from Calvitti et al. (Calvitti et al. 2015) where the CI is $100\%$ effective for *w*AlbAB males in $M_{\textit{w}AlbAB, 1}$–$M_{\textit{w}AlbAB, 14}$ (that is, no viable offspring), $67\%$ effective for *w*AlbAB males in $M_{\textit{w}AlbAB, 15}$–$M_{\textit{w}AlbAB, 19}$, and $0\%$ effective for *w*AlbAB males in $M_{\textit{w}AlbAB, 20}$ (that is, no CI preventing offspring). Along with increased fitness (Moretti and Calvitti 2013), this gives the AR*w*P mosquitoes a slight reproductive advantage over the wild-type *w*AlbAB mosquitoes. It is important to note that the compartments $M_{w, j}$, for *Wolbachia* strain *w* and $j=1, 2,\dots , K$, do not capture the age of the mosquito, but rather the distribution of time a mosquito will spend with a given level of CI. That is, in our model, adult mosquitoes have an exponentially distributed lifespan, but a truncated Gamma-distributed time spent with a given CI efficacy.

Another feature of our model is the inclusion of immigration and emigration. Although we are only investigating a single-patch model, the patch represents a city block of urban houses. City blocks do not exist in isolation, and this may have consequential effects on any suppression control programs being examined. We assume that all neighbouring patches, not explicitly modelled, are in an equilibrium steady state for the wild-type *w*AlbAB mosquitoes and that AR*w*P releases are only occurring on our modelled patch. Hence, all immigrant mosquitoes are of the wild-type. However, we allow both the wild-type and the invasive AR*w*P to emigrate out of the modelled patch, noting that the probability that an AR*w*P mosquito emigrates out of the modelled patch and then immigrates back is small and will have negligible impact. Immigration and emigration rates are calculated such that they leave a net-zero change in the steady-state wild-type population; see the Supplementary Material for more details.

Our model is biologically feasible when $\frac{\mu _F}{\frac{1}{2} \lambda p_{mated}} < 1$ (that is, when females give birth to new generations of females before they die), where $\mu _F$ is the female death rate, $\lambda$ is the base birth rate of future adult mosquitoes, and $p_{mated}$ is the proportion of wild-type female mosquitoes that are mated in the steady-state equilibrium; see the Supplementary Material for the derivation of this constraint. This constraint means that point estimates of female death rates of 8–9 day life spans (Vavassori et al. 2019) are too short to be feasible in our model. As such, in all investigations in this paper, we have derived the female death rate ($\mu _F$) as the largest biologically feasible value satisfying $\frac{\mu _F}{\frac{1}{2} \lambda p_{mated}}=0.999$ for fixed $p_{mated}$ and $\lambda$. Note, the values for $\mu _F$ suggest an average female life span of 9 to 10 days (approximately 1 day longer than existing point estimates), well within biologically feasible ranges (Vavassori et al. 2019).

A generalised version of this model for *J* strains of *Wolbachia* is presented in the Supplementary Materials.

### Simulation studies

We perform two separate simulation studies. The first investigates the unstable equilibrium threshold for when the AR*w*P-infected *Ae. albopictus* are likely to establish in the wild-type population. The second investigates the effects of immigration and emigration for three different IIT release strategies of AR*w*P-infected *Ae. albopictus* into the wild-type population. All simulations were run for the expected, low, and high fitness parameter values (see Supplementary Materials Table S1) to explore sensitivity of the results. We also investigate sensitivity to the age-decay of CI, and reductions in mating competitiveness based on values from recent field trials (Caputo et al. 2023).

The code was run on a high performance computing Dell PowerEdge C6525 Linux cluster, running for 72 h over 20 CPU cores. All code was written in R v 4.5.1 and is available at https://github.com/Matthew-Ryan1995/bidirectional_IIT_control_model.

#### Cage simulations

A major concern with IIT control programs is the establishment of the artificial *Wolbachia* strain in the wild mosquito population, in this case *Ae. albopictus* infected with AR*w*P. To mitigate this concern, IIT control programs aim to keep the artificial strain under an unstable equilibrium threshold (or probability) of establishment, which we denote by $\omega ^*$. The value of $\omega ^*$ is defined such that if the proportion of the artificial *Wolbachia* strain (*Ae. albopictus* infected with AR*w*P) relative to the initial wild-type population (*Ae. albopictus* infected with *w*AlbAB) exceeds $\omega ^*$, then the artificial strain is more likely to establish.

The unstable equilibrium threshold of establishment $\omega ^*$ has been studied for *Ae. aegypti* in the context of a uni-directional CI IIT control program (Axford et al. 2016). Axford *et al.* found through cage experiments that $\omega ^*$ is between 0.20 and 0.25 for *Ae. aegypti* when the invasive *Ae. aegypti* are infected with *w*AlbAB (Axford et al. 2016), which has been validated *in silico* (Pagendam et al. 2020). However, there is no *a priori* reason to expect that $\omega ^*$ will remain unchanged in a bi-directional CI IIT control program. In fact, Moretti et al. (2018) suggest that the unstable equilibrium threshold is inappropriate for *Ae. albopictus*. Further, Lombardi et al. (2024) recently conducted cage experiments for *Ae. albopictus* to consider the effects of bi-directional CI on IIT control programs and found an unstable equilibrium of approximately $\omega ^* = 0.40$ (although some of these experiments resulted in establishment). We aim to investigate these results further using *in silico* cage experiments.

Here, we investigate feasible values for $\omega ^*$ in the context of a bi-directional CI IIT control program for *Ae. albopictus* using *in silico* cage simulations. As qualitative controls, we also investigate values of $\omega ^*$ when CI is uni-directional and when CI is bi-directional but without age-related decay. Our primary scenario starts with an initial *Ae. albopictus* adult mosquito population of 420, equally split between males and females, and varies the initial proportion of mosquitoes infected with AR*w*P from 0.05 to 0.50 by increments of 0.05. As a qualitative control for these cage simulations, we compare our uni-directional results with those from the Pagendam et al. (2020) model, which were in turn directly compared with experimental findings. These qualitative control simulations are equivalent to a wild-type *Ae. albopictus* population which has been cleared of *Wolbachia*. For each initial proportion of AR*w*P, we run $1\ 000$ simulations.

Simulations were run for up to 500 simulation days or for 180 days after one of the following two stopping conditions were met: 1) the number of AR*w*P adults reached zero or 2) the adult *Ae. albopictus* (either *w*AlbAB or cleared) population was suppressed, where suppression is defined as the population dropping below $10\%$ of the initial population (that is, when the number of adult *Ae. albopictus* mosquitoes dropped below 42). A $10\%$ initial population criteria is more conservative than the $30\%$ population suppression threshold used by Fonseca et al. (2013).

#### Management decisions: IIT release scenarios

We examined three different IIT release scenarios and the effects of different immigration and emigration rates on the efficacy of these control programs. Each of the IIT release scenarios followed the same basic principles, but differed on when the release was terminated. The basic scenario was as follows: every seven days, AR*w*P mosquitoes will be released into the patch at an overflooding ratio of 5 : 1 (Lombardi et al. 2024; Pagendam et al. 2020; Moretti et al. 2018), meaning that the number of *Wolbachia*-infected males released into the population at each event was five times the current wild-type male population size. We assume the number of wild-type male mosquitoes is known at each release time. A female contamination rate of 0.01 was used for the releases (Moretti et al. 2018; Mamaï et al. 2024) with scenarios running for at least 100 days before any stopping condition was considered. The three stopping conditions were:

- **Naïve scenario**: *Wolbachia* release will run until the adult *w*AlbAB population drops below $10\%$ of the initial steady-state population.
- **Complete stop scenario:** *Wolbachia* release will run until the adult *w*AlbAB population drops below $10\%$ of the initial steady-state population *OR* the proportion of AR*w*P adults exceeds the unstable equilibrium threshold $\omega ^*$.
- **Maintain scenario:** *Wolbachia* release will run until the adult *w*AlbAB population drops below $10\%$ of the initial steady-state population *OR* the proportion of AR*w*P adults exceeds the unstable equilibrium threshold $\omega ^*$. Releases will resume if the proportion of *w*AlbAB adults returns to more than $10\%$ of the initial population (that is, they re-establish) **AND** the proportion of AR*w*P adults drops below $0.8 \omega ^*$.

The naïve scenario represents the simplest and most obvious baseline to achieve suppression of the wild-type population. The complete stop scenario explores a cautious implementation of a dual objective scenario of suppressing the *w*AlbAB mosquito and preventing the artificial *Wolbachia* strain from establishing by stopping releases at the unstable equilibrium threshold $\omega ^*$. The maintain scenario explores achieving these dual objectives, whilst continuing the release of AR*w*P under sustainable conditions (avoiding establishment: the second objective). Note that the condition for restarting releases when the proportion of AR*w*P drops below $0.8\omega ^*$ was chosen for convenience, to prevent stop-start oscillations from having identical threshold values.

To investigate the extent that immigration and emigration will affect these release scenarios, we consider three mosquito immigration rates. The rates chosen represent i) a closed, isolated population with no immigration, ii) a low immigration/emigration rate (2 adults per week), and iii) a high immigration/emigration rate (10 adults per week).

For each release scenario and immigration/emigration rate, we ran $1\,000$ simulations. Simulations were run for 920 simulation days (2.5 years), with AR*w*P releases occurring until the stopping condition was met or for a maximum of 730 days (2 years). We ran simulations for 6 months after the cessation of all release scenarios to investigate the reversibility of the IIT control program. At the end of the simulations, we determined the proportion of successful scenarios defined as suppressing the *w*AlbAB population below $10\%$ of the initial population size while keeping the invasive AR*w*P below the unstable equilibrium threshold $\omega ^*$. We compared the proportion of successful scenarios at the end of the release strategy and again 6 months after to assess the scenario’s longevity and reversibility. Additionally, we calculated a proxy for the cost of the release scenario based on the number of adult mosquitoes released during the lifetime of the simulation. Per insect measures are a field standard cost proxy that can allow overheads and rearing costs to be translated to different contexts (Soh et al. 2021; Baly et al. 2025; Scott et al. 2017).

## Results

### Cage simulations

**Fig. 2:**
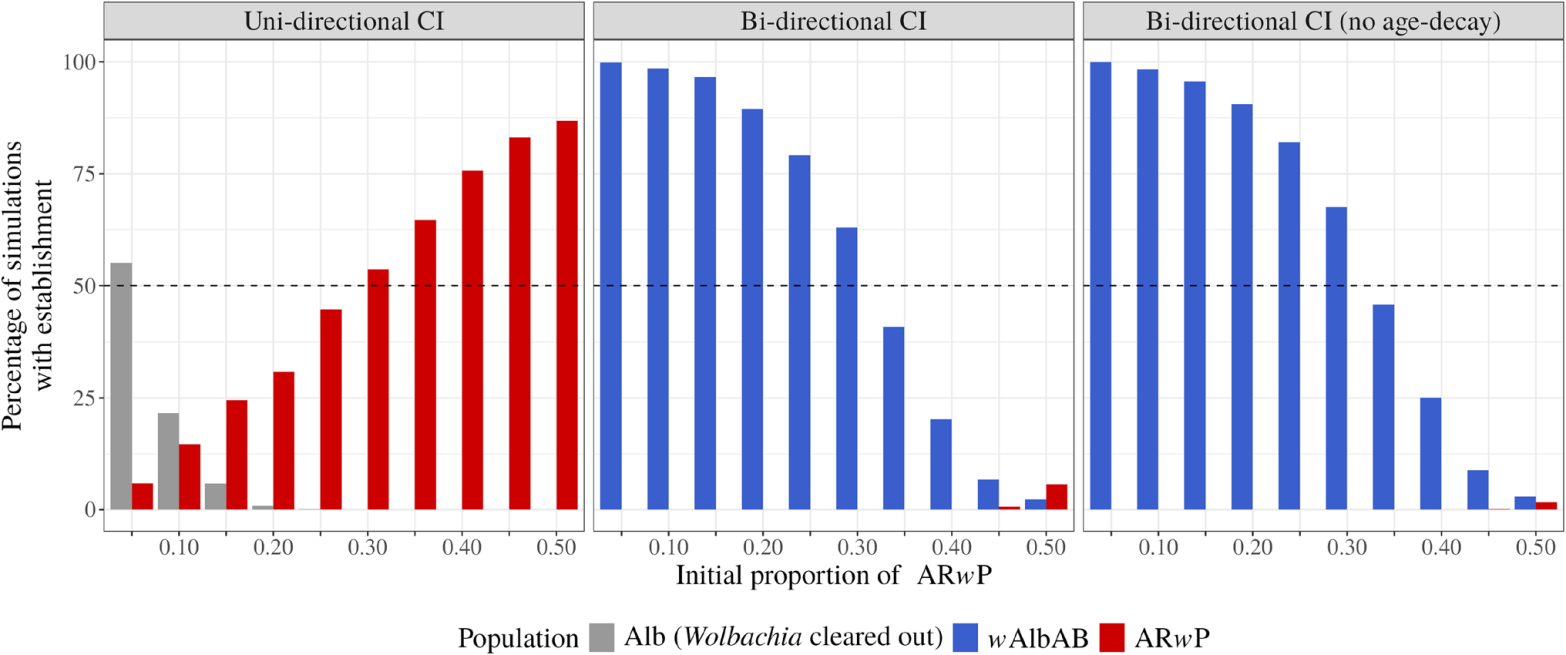
Percentage of *in silico* cage simulations ending in population establishment at the end of the 500 iterations. An *Ae. albopictus* population is considered established if the proportion of adults is greater than $10\%$ of the initial population size. The left panel shows simulations with uni-directional cytoplasmic incompatibility (CI), the middle panel depicts bi-directional CI with age-decay and the right panel bi-directional CI without age-decay. Red columns represent the AR*w*P population, blue columns the *w*AlbAB population, and grey columns the *Wolbachia* cleared (does not contain *w*AlbAB) *Ae. albopictus* population. The *X*-axis indicates the initial proportion of AR*w*P, and the *Y*-axis shows the percentage of simulations that resulted in establishment. The dotted horizontal line marks $50\%$ of simulations. Each simulation started with 420 adult mosquitoes (half male and half female), and the fitness parameters are the expected values defined in Supplementary Materials Table S1

We found a higher unstable equilibrium threshold for *Ae. albopictus* than *Ae. aegypti* when only considering uni-directional CI (Pagendam et al. 2020; Axford et al. 2016). That is, Pagendam et al. (2020) found for *Ae. aegypti* that when the frequency of adults with a *Wolbachia* infection starts at $25\%$ of the initial population the *Wolbachia* strain will establish in at least $50\%$ of the simulations; for *Ae. albopictus*, we found the invasive strain needs to start at $30\%$ of the initial population to have the same property (where the red columns meet the horizontal dashed line, left panel, Fig. 2).

In contrast, the results for the bi-directional CI case are striking (middle panel, Fig. 2). Our simulations showed that at least $45\%$ of the initial population is required to start as AR*w*P for simulations to result in an outcome where the artificial *Wolbachia* strain established (red columns, middle panel, Fig. 2). This bi-directional CI result is driven by AR*w*P failing to find compatible mates for smaller population sizes and are consequently driven to extinction by density effects. Based on these results, we consider an unstable equilibrium threshold as $\omega ^* = 0.4$, the cut-off to prevent AR*w*P from becoming established in the wild. These results are qualitatively consistent for the high and low fitness parameters (Figs. S7-S8).

When removing age-related decay of CI in the bi-directional simulations, the same threshold $\omega ^*=0.4$ is suggested (Fig. 2, right panel). However, there are qualitative differences in the likelihood of AR*w*P establishment across the two bi-directional scenarios. When CI decays with mosquito age, AR*w*P is more likely to establish in the *in silico* cage population for initial proportions of AR*w*P greater than 0.4. The effect of age-related decay is more pronounced for the extreme fitness parameters, particularly at the $50\%$ relative abundance threshold (Figs. S7-S8).

Reducing mating competitiveness suggests the threshold $\omega ^* = 0.4$ is robust, with the possibility of optimising releases for a higher threshold if the Fried’s index for a specific locale is known (Fig. S2). We also observe qualitative differences in the suppression of the wild-type population, indicating suppression is more difficult with reduced mating competitiveness.

### Comparison of IIT release scenarios

**Fig. 3:**
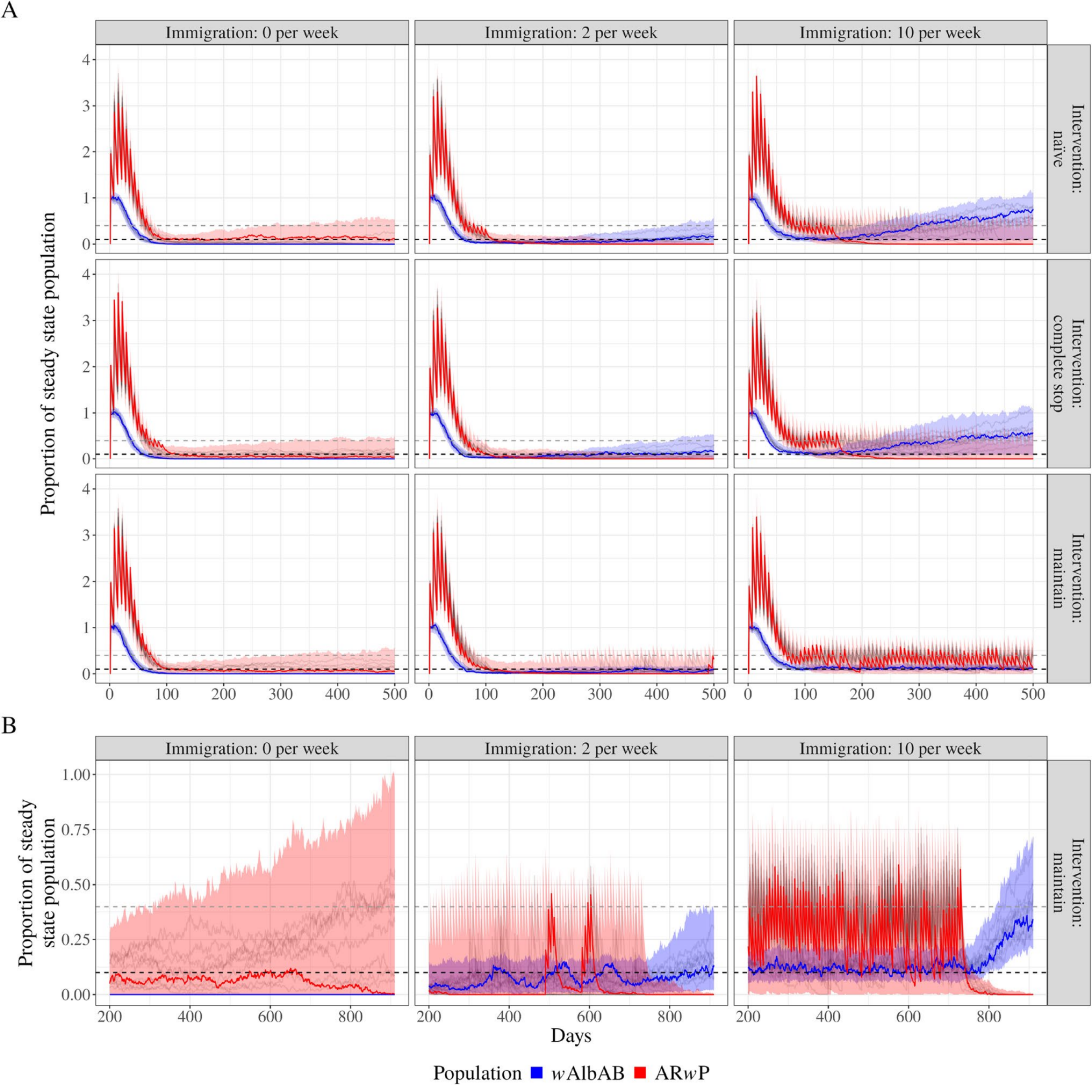
Proportion of mosquitoes relative to the steady-state population over time for different release scenarios (rows) and immigration rates (columns). The AR*w*P population (red) and *w*AlbAB population (blue) lines represent the median with $95\%$ confidence intervals (shaded areas). Black lines represent 10 random simulation runs. Panel A displays the population over 500 days under three immigration rates (0, 2, and 10 mosquitoes per week). The intervention scenarios include naïve, complete, and maintain. Panel B focuses on the maintain scenario over days 200 to 920 for the same immigration rates. Horizontal-dotted lines represent the suppression/establishment ($10\%$) and the unstable equilibrium ($40\%$) thresholds used. Values above 1 on the Y-axis indicate population levels higher than the initial steady state. Model parameters are the expected values defined in Supplementary Materials Table S1

The maintain scenario was the suppression strategy most likely to reflect the control of a mosquito population using the IIT in the real world. Here the rate of immigration and emigration are important as they increase the wild *Ae. albopictus* population, influencing the decision to resume or maintain releases of AR*w*P infected males. There were stark contrasts between the immigration rates under a maintain scenario (Fig. 3 B). High immigration, when compared to low immigration rates, influences the total number of released AR*w*P-infected males required to suppress the wild population and also increases the total size of both established *Wolbachia* populations. This suggests that it may be difficult to maintain suppression on a wild mosquito population with high immigration rates. In contrast, a low immigration rate (2 adults per week) leads to an extended period of low growth after suppression in the wild *Ae. albopictus* population (Fig. 3 A). In our low immigration, maintain scenario this occurred between approximately 120 and 220 days, after which AR*w*P releases restarted due to the wild *Ae. albopictus* population increasing to $10\%$ of its original size.

There is no qualitative difference between the naïve and complete stop release scenarios for all immigration rates (first two rows, Fig. 3 A). The naïve scenario resulted in the recovery of the wild *w*AlbAB population after releases ceased under low and high immigration rates (Fig. 3 A, top row). Likewise, wild-type populations recovered when AR*w*P releases ceased during the complete stop scenarios with low and high immigration (Fig. 3 A, middle row). Variation in the rate of wild *Ae. albopictus* population recovery in both the naïve and complete stop scenarios was dependent upon total immigration/emigration, with high immigration rates leading to an almost complete rebound after 500 days in the majority of simulations. Interestingly, under low immigration and both the naïve and complete stop scenarios, the wild population was less than $40\%$ of its initial population ($<168$ adult mosquitoes) for the majority of simulations after 500 days, indicating a slow rebound for the wild-type population.

Low levels of AR*w*P replacement did occur across all release scenarios with no immigration, even after suppression was successful and releases ceased (Fig. 3 A, first column). This is the result of low levels of female contamination introduced during the overflooding stage of the first 100 days of scenarios.

These results are consistent across the high and low fitness parameters, with the caveat that there is no qualitative difference between the three strategies when immigration is low for the high fitness parameters (Figs. S9 and S10).

When age-related decay of CI is removed from the bi-directional model, there are slight qualitative differences in the results (Fig. S14). Specifically, when immigration is high in the naïve scenario, the expected time to suppression of the wild-type *w*AlbAB population is longer, requiring more releases of the invasive AR*w*P strain on average.

Greatly reducing mating competitiveness shows qualitatively similar results for no and low immigration rates but has notable differences for high immigration (Fig. S3), particularly for the naïve and complete stop interventions. Specifically, suppressing the wild-type mosquito becomes difficult when mating competitiveness is low and immigration rates are high, requiring longer release periods. This low mating competitiveness and high immigration scenario indicated a reduced capacity for the AR*w*P mosquitoes to mate and produce sterile offspring. Reducing mating competitiveness a moderate amount results in qualitatively similar results (Fig. S4).

### Success of IIT release scenarios

The AR*w*P population is kept below $40\%$ of the initial population size for all IIT release scenarios and all immigration rates, both at the time release is stopped and six months after (Table 1), indicating successful outcomes. Within 6 months of releases ceasing the AR*w*P population is kept below 168 mosquitoes across all simulations, likely due to lack of viable mates. For the *w*AlbAB population, IIT release scenarios are successful for all immigration rates and release scenarios, except for the maintain scenario when immigration is high (Table 1). Since releases are ongoing and immigration is high in this scenario, the *w*AlbAB population is kept above 42 in more than $88\%$ of simulations. Six months after releases of AR*w*P mosquitoes are ceased, we observe evidence of reversibility of the IIT control program when immigration is low, and complete reversibility when immigration is high.

These results are qualitatively similar for the low fitness parameters (Table S4). For the high fitness parameters, the only scenario that successfully suppresses the *w*AlbAB population and keeps the AR*w*P population below the unstable equilibrium threshold is the naïve scenario under no or low immigration rates. However, for these parameters, all scenarios are successful within 6 months of ceasing releases except when immigration is high, where IIT release scenarios are reversible for the wild-type *w*AlbAB population (Table S5). When age-decay of CI is removed from the model, there is a slight reduction in management success of the *w*AlbAB population after 6 months for low immigration rates (Supplementary Materials Table S6). Mild reductions in mating competitiveness show qualitatively similar results in management success, but when mating competitiveness is low we observe a substantial reduction in the success of the interventions (Table S3).

**Table 1:** Percentage of simulations with successful outcomes for *w*AlbAB and AR*w*P populations under different immigration rates and release scenarios

	Naïve		Complete stop		Maintain	
Scenario	*w*AlbAB	AR*w*P	*w*AlbAB	AR*w*P	*w*AlbAB	AR*w*P
*0 Mosquitoes per week*						
Within stopping time	100.0	100.0	100.0	100.0	100.0	100.0
After 6 months	100.0	99.9	100.0	100.0	100.0	100.0
*2 Mosquitoes per week*						
Within stopping time	100.0	100.0	100.0	100.0	92.2	100.0
After 6 months	81.8	100.0	80.1	100.0	52.2	100.0
*10 Mosquitoes per week*						
Within stopping time	99.8	100.0	99.7	99.7	21.2	100
After 6 months	0.0	100.0	0.0	100.0	0.0	100.0

A successful outcome is defined as keeping the *w*AlbAB population below $10\%$ of the initial population size, and AR*w*P below $40\%$ (unstable equilibrium threshold). The data are shown both within the stopping time and six months after

### “Cost” of an IIT control program

**Fig. 4:**
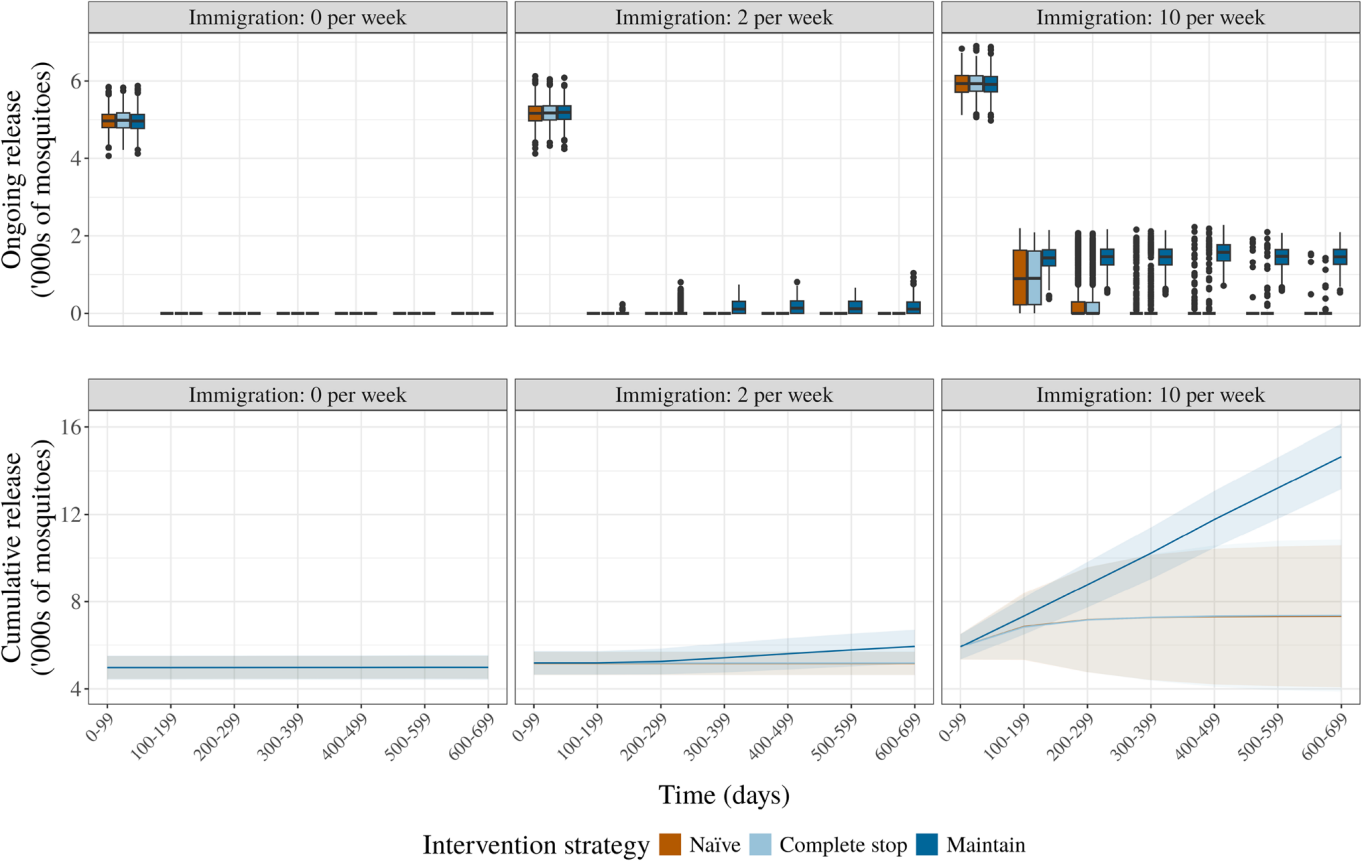
The release in thousands of AR*w*P mosquitoes as a proxy for cost under different intervention strategies and immigration rates. The *X*-axis represents the time in 100-day intervals up until day 700. The top row shows the ongoing cost every 100 days, measured as the number of mosquitoes released every 100 days for each intervention strategy (naïve, complete stop, and maintain). The bottom row illustrates the cumulative release (in thousands of mosquitoes) over the entire period for each strategy. The model parameters are the expected values defined in Supplementary Materials Table S1

We compare the number of mosquitoes released for each scenario and immigration rate to compare different release scenarios (Fig. 4). All release scenarios have a consistent cost in the first 100 days of releases. Note that this is by design as all three scenarios run for at least 100 days before considering any stopping conditions. The no immigration scenario acts as a comparison, with approximately 5,000 adult mosquitoes released before ceasing at the 100-day interval. Once the wild mosquito population is suppressed in the low immigration scenario, it is only the maintain intervention strategy that continues to accumulate further adult releases, though these are in the hundreds of adults per week only and total approximately 6, 000 adults after 700 days (Fig. 4, middle column). When immigration is high, adult male AR*w*P releases need to match the wild immigration rate to maintain suppression on the *Ae. albopictus* population. For the maintain intervention strategy, this overflooding rate increases linearly through time, accumulating to over 14, 000 adults over 700 days and is the most expensive of all strategies proposed.

Despite the increased ongoing cost of the maintain scenario, the results are more predictable. In contrast, the naïve and complete stop scenarios have a higher variance. These results are qualitatively consistent for the low fitness parameters (Fig. S12). For the high fitness parameters, the naïve scenario has the greatest ongoing and cumulative cost due to the increased difficulty in suppressing the wild *w*AlbAB *Ae. albopictus* population (Fig. S13). When age-related decay of CI is removed in the bi-directional model, there are no qualitative differences in the estimated cost of each scenario (Fig. S14).

The number of mosquitoes released is qualitatively similar for greatly reduced mating competitiveness for no or low immigration, but different for high immigration rates (Fig. S5). As expected from the increased difficulty to suppress the wild-type population, when immigration is high costs scale similarly across all three release strategies. Costs are qualitatively similar for moderate mating competitiveness (Fig. S6)

## Discussion

As the incompatible insect technique continues to be successfully deployed across the world (Gaudillat et al. 2025; Li et al. 2025; Caputo et al. 2023), there is an increasing need to explore novel management strategies which suppress medically important mosquito populations. These strategies are broadening in scope to include complex landscapes and species with novel *Wolbachia* strains and bi-directional CI effects. Traditionally, the release of females of an artificially-infected *Wolbachia* strain through imperfect sex separation would jeopardise the effectiveness of an IIT control program. However, when wild mosquito populations also exhibit a CI phenotype, we demonstrate, *in silico*, how suppression and establishment of an artificial *Wolbachia* strain is inhibited by immigration from a wild population.

The Markov population process model extended here (from Pagendam et al. 2020) allows for increasing complexity of both landscape and biological parameters, modelled stochastically around *Wolbachia* establishment thresholds. Where previous authors have concerned themselves with questions around uncertainty of establishment through low levels of female contamination (Soh et al. 2022; Pagendam et al. 2020; Matsufuji and Seirin-Lee 2023), here, we have intentionally explored the release of artificially-infected *Wolbachia* female mosquitoes to understand the stability of establishment under a bi-directional CI system. Our approach also explored the inclusion of age-related decay of CI for adult male mosquitoes. Importantly, our results highlight the attributes of *Wolbachia* IIT control programs that seek to maintain a wild *w*AlbAB *Ae. albopictus* mosquito population under a high level of suppression while maintaining a manageable level of AR*w*P *Wolbachia* establishment. These include: (i) higher AR*w*P invasion thresholds due to bi-directional CI impacting the efficiency of *Wolbachia* establishment and (ii) release strategies that consider rates of wild mosquito population immigration into treatment blocks, with higher levels of immigration impacting both growth of the wild mosquito population and the efficiency of AR*w*P establishment. Attribute (i) highlights the flexibility a bi-directional CI system has on the success and failure of an IIT control program, while attribute (ii) starts to highlight the complex nature of biological systems, with immigration and emigration impacting outcomes of different release strategies.

We estimate a new unstable equilibrium threshold for establishment in a bi-directional, AR*w*P *Wolbachia* system as approximately $40\%$ of the original population, smaller than the $50\%$ suggested by Moretti et al. (Moretti et al. 2018). Interestingly, the same threshold for AR*w*P establishment is suggested when age-decay of CI is removed from the model. Our threshold is a conservative estimate, as only a small percentage ($0.7\%$) of simulations led to establishment at the 0.45 initial proportion of AR*w*P (Fig. 2). Our findings align with those observed in bi-directional CI IIT cage studies undertaken by Lombardi et al. (2024) which observed a slight decrease in the infection of AR*w*P infected females at approximately $40\%$ of the caged population. At close to double the establishment threshold of $22\%$ in an *Ae. aegypti* uni-directional system (Axford et al. 2016; Pagendam et al. 2020), we suggest our estimated *Ae. albopictus* threshold is primarily the result of inefficient mating (that is, adults of each *Wolbachia* strain failing to find a compatible mate) in a well-mixed population. This observation is also based on differences in mosquito biology (e.g. fitness, average life expectancy, and CI changes over time) between *Ae. aegypti* and *Ae. albopictus*. These *in silico* simulations provided the establishment threshold for the intervention scenarios developed herein, which take into consideration immigration/emigration, female contamination rate and male release scenarios of an artificial *Wolbachia* strain.

The stable maintenance of population suppression is the primary goal of any mosquito IIT control strategy. Three management scenarios were chosen to represent different control decisions, primarily focused on whether AR*w*P releases continue or are ceased. Results demonstrate the logical end points of scenarios where: AR*w*P replacement occurs (no immigration, all three management scenarios); the wild-type population is allowed to recover (low and high immigration, naïve and complete stop scenarios); or ongoing management controlled both mosquito populations (low and high immigration, maintain scenarios).

When we considered isolated populations with no immigration, we observed low levels of AR*w*P establishment across all management. This is consistent with previous modelling for *Ae. aegypti* (Pagendam et al. 2020) and *Ae. albopictus* (Moretti et al. 2018) which suggests that even under high levels of suppression in an isolated population, establishment may be stochastically driven. There was no qualitative difference between the complete stop and naïve release strategies due to their shared stopping criteria, but both scenarios showed evidence of reversibility of the IIT control program in the presence of immigration of the wild-type strain. These results agree with previous modelling by Moretti et al. (2018), but our model suggests an even lower immigration rate may be sufficient for a population rebound post-release termination. Finally, the maintain scenario was the most effective at maintaining a stable wild-type population in the presence of immigration, reflecting a likely management scenario in the real world as recently conducted across islands in China (Li et al. 2025). In their experiments, Li et al. (2025) found their bi-directional IIT control program was reversible upon stopping release of the invasive *Wolbachia* strain, a result echoed by our modelling. While not simulated here, scaled interventions would likely cover larger urban areas, with multiple patches the size of our single population. Due to the strong effect of immigration rates on continual release and cost, these results suggest an *Ae. albopictus* bi-directional CI intervention should aim to reduce immigration between patches to something more easily manageable across the entire area being treated, a result echoed in *Ae. aegypti* management programs (Consortium and Ching 2021; Montenegro et al. 2024; Crawford et al. 2020).

IIT control programs need to consider the cost of male delivery to ensure the programs are competitive with traditional insecticide-based approaches. Here, we model total mosquitoes as a proxy for cost, noting this ignores potential context dependent fixed overheads or costs related to rearing, sorting and labour to deliver individual male mosquitoes into a landscape. It is clear from the total mosquito economic proxy that cost scales linearly for a small population with a carrying capacity of 420 mosquitoes, and that it is relatively expensive to suppress a population that is constantly receiving a large weekly migration ($>2.5\%$ of the population) of individuals from outside the release area. Again, these results suggest that a low immigration rate between populations would reduce costs. We hypothesise that the effect of reduced immigration on cost is caused by the relative abundance of each *Wolbachia* strain being approximately $50\%$ (Fig. 2), causing a reduction in mating efficiency of adults finding compatible mates in a well-mixed population. However, the mating efficiency when considering competing *Wolbachia* strains has not yet been explored in low abundance or densities.

Our results are robust to removing the age-related decay of CI in the *w*AlbAB population. This suggests that the decay of CI of *w*AlbAB mosquitoes does not substantially impact IIT control programs for these population sizes and modelled scenarios. However, our *in silico* cage experiments suggest that age-related CI decay may play a key role in mating efficiencies when the relative abundance of each *Wolbachia* strain is $50\%$ (Fig. 2) or when considering larger populations (Fig. S8). Additionally, there are some nuanced qualitative differences in the control of *w*AlbAB populations over the two year period modelled here (Fig. S14). Further research is needed to determine the effect of age-decay of CI on real-world IIT control programs. In particular, the interactions of age-related CI decay with seasonality, populations experiencing mass migration, or larger population sizes provide interesting areas for further study.

We investigated the sensitivity of our results to extremes in fitness parameters and reductions in mating competitiveness. For low fitness parameters, that is, smaller populations with shorter lifespans, our results are qualitatively consistent. However, in larger populations with longer lifespans, the wild-type population may be more difficult to suppress if immigration rates are high (Fig. S10). Reducing mating competitiveness for the invasive AR*w*P mosquitoes shows that our equilibrium threshold of $40\%$ holds but has scope to be optimised for specific contexts (Fig. S2). Optimising the equilibrium threshold may be critical for the success of real-world field trials, since mating competitiveness may be as low as $36\%$ (Caputo et al. 2023). When mating competitiveness is this low population replacement can be successfully avoided even with a higher threshold. However, if immigration is high, reduced mating competitiveness may make population suppression difficult (Fig. S3). Together, these indicate that improved estimates of mating competitiveness are needed for AR*w*P, as well as improved understanding of mosquito movement, to help guide successful implementations of the IIT. Further sensitivity analyses are computationally prohibitive for this stochastic model.

As a first conceptual model towards understanding these complex IIT releases, there are a number of limitations and opportunities for future work. Despite consideration of a complex model of the mosquito life cycle, we made simplifying assumptions about fitness parameters by explicitly excluding seasonality and overwintering, which might be important depending on climactic context (Kramer et al. 2021). Future work may investigate the impacts of these drivers of mosquito populations on IIT control programs, but other modelling approaches (Griffin et al. 2023) or different models of *Ae. albopictus* life cycles (Erickson et al. 2010) may be more appropriate. We modelled simple release strategies every 7 days, based on knowledge of the current mosquito population for an overflooding approach and a single modelled spatial location (or patch). However, the future success of the IIT relies on the critical need for the technology to scale efficiently and effectively over large areas. These findings should be modelled over a larger, meta-population scale typical of a large intervention. In such a scenario, we expect implementing IIT releases in multiple patches will have strong consequences for program effectiveness, reversibility, and the impacts of immigration and emigration. Further, the interventions considered should be formally optimised to the desired outcomes of the program under cost and time requirements. When considering a meta-population approach, this optimisation will include identifying optimal spatial locations to manage release strategies, which has recently been considered for IIT control programs for *Ae. aegypti* in Singapore (Lim et al. 2024). If the objective is to reduce transmission, this will require a transmission model or consideration of vectorial capacity, a feature that is not considered here. Finally, our suppression criteria of $10\%$ of the original population represents a conservative value based off a recommendation for a control program reducing nuisance biting in a North American context (Fonseca et al. 2013). Future estimates of this criteria should consider how high levels of suppression on a mosquito population impacts the vectorial capacity of a species and its ability to transmit pathogens between human hosts.

## Conclusions

Robust planning and evidence-based science is essential when following the principle of “*Primum non nocere*” or “first, do no harm” when trialling new technologies. One condition communities and regulators have insisted on for pioneering population suppression technologies such as *Wolbachia* IIT and genetic engineering approaches is the ability to return a wild insect population to an original state. The release of females through imperfect sex-separation technologies has been considered an unwanted outcome of the IIT in the past. However, we suggest that immigration of a wild *Wolbachia* strain (*w*AlbAB) can be used to suppress the establishment of an artificially introduced *Wolbachia* strain (AR*w*P). Further, we have demonstrated that the introduced strain can be eradicated if immigrants (or inflows of the original strain) are utilised to return the targeted insect population to a previous natural state. This was a common outcome in all management scenarios where immigration was present and *Wolbachia* (AR*w*P) releases had ceased; the higher the immigration rate, the quicker the population approached its original natural steady state. Returning an insect population to a previous natural state is an outcome that may be desirable by local communities opting out of a *Wolbachia* program, or if a commercial program is no longer financially viable.

## Author contributions

MR contributed to methodology, software, validation, formal analysis, investigation, writing—original draft, writing—review and editing, and visualisation. MM contributed to software, validation, formal analysis, investigation, writing—original draft, writing—review and editing, and visualisation. DP contributed to conceptualisation, methodology, software, and writing—review and editing. RH contributed to conceptualisation, methodology, software, validation, formal analysis, writing—original draft, writing—review and editing, and visualisation. BT contributed to conceptualisation, methodology, validation, formal analysis, writing—original draft, writing—review and editing, visualisation, and funding acquisition.

## Supplementary Information

Below is the link to the electronic supplementary material.

Supplementary file 1 (pdf 21629 KB)

## Data Availability

The code is available at https://github.com/Matthew-Ryan1995/bidirectional_IIT_control_model. The only data used was parameter values, available from both Table S1 and the code repository.
