# Supplementary material for: Modelling Aedes albopictus management, incorporating immigration and bi-directional Wolbachia interactions
Source: J Pest Sci (2004). 2026 Mar 10;99(2):58. doi: 10.1007/s10340-026-02030-4 (PMC12975787; doi:10.1007/s10340-026-02030-4)
Supplement: Supplementary file 1 — (pdf 21629 KB) [file 10340_2026_2030_MOESM1_ESM.pdf]

Supplement to Modelling *Aedes albopictus* management, incorporating immigration and bi-directional *Wolbachia* interactions

1 Model details

Here the full model details are described, with Table S1 outlining the model parameters and values, and Table S2 providing the full model transitions for the continuous time Markov chain (CTMC) implementation of our Markov population process. When referring to "high fitness parameters" the upper bounds for the number of immature classes, the proportion of mated females and the carrying capacity were used, and the lower bounds for the birth and death rates were used (Table S1, note the markers). This choice for high fitness parameters means the mosquitoes live longer lives in larger populations. For "low fitness parameters" the other extrema were used, such that the mosquito lives were on average shorter and populations smaller.

Table S1: Descriptions and values of model parameters. All rates are in units of per day unless otherwise specified. † high fitness parameters. ♭ low fitness parameters. CI - Cytoplasmic incompatibility.

Symbol	Description	Value (min, max)	Source
K	Number of male classes; average number of days male CI changes	20	[3]
σ	CI changing rate	$1/K$	
k	Number of immature classes; average number of days for an egg to mature into an adult	12 ($10^\flat$, $50^\dagger$)	[1]
γ	Maturation rate of immature classes	$1/k$	
p_{mated}	Proportion of females mated at steady state	0.800 ($0.500^\flat$, $0.800^\dagger$)	
C	Total adult population at steady state	420 ($400^\flat$, $800^\dagger$)	[5]
λ	Intrinsic birth rate of females	0.253 ($0.106^\dagger$, $0.400^\flat$)	[2, 6]
p_f	Proportion of immatures that become adult females	0.500	
p_m	Proportion of immatures that become adult males	$1 - p_f$	
μ_M	Death rate of adult males	0.128 ($0.043^\dagger$, $0.230^\flat$)	[8]

Continued on next page

Table S1 – *Continued from previous page*

Symbol	Description	Value (min, max)	Source
μ_F	Death rate of adult females	0.100 (0.041†, 0.099$^{\flat}$)	Eq (S34)
$\zeta^F = \zeta^M$	Overall male/female immigration rate	0.286 (0, 1.429)	
ξ^M, ξ^F	Emigration rates	Eq (S27)	
$I_{\max}$	Carrying capacity of immatures	Eq (S17)	[5]
η	Mating rate between adults	Eq (S18)	
$c_{wAlbAB\times ARwP,l}$	CI leading to proportion of viable offspring by mated *w*AlbAB females with AR*w*P males of age l	1 $\forall l$	[3]
$c_{ARwP\times wAlbAB,l}$	CI leading to proportion of viable offspring by mated AR*w*P females with *w*AlbAB males of age l	1 (l = 1–14), 0.68 (l = 15–19), 0 ($l \geq 20$)	[3]
$\text{Fried}_{wAlbAB}, \text{Fried}_{ARwP}$	Fried's index for AR*w*P and *w*AlbAB.	1	

Table S2: Stoichiometries of the full model.

Event	State change	Transition rates
Birth of an immature	$I_{w,1} + 1$	$\tilde{\lambda}\bar{F}_w$
Ageing of immatures	$(I_{w,i}, I_{w,i+1}) + (-1, 1)$	$k\gamma I_{w,i}, \forall i \in [1, k-1]$
Maturation into male	$(I_{w,k}, M_{w,1}) + (-1, 1)$	$p_m k\gamma I_{w,k}$
Maturation into female	$(I_{w,k}, F_w) + (-1, 1)$	$p_f k\gamma I_{w,k}$
Ageing of male	$(M_{w,i}, M_{w,i+1}) + (-1, 1)$	$K\sigma M_{w,i}$
Death of male	$M_{w,i} - 1$	$\mu_M M_{w,i}$
Death of unmated female	$F_w - 1$	$\mu_F F_w$
Mating	$(F_w, F_{w\times v,l}) + (-1, 1)$	$\eta F_w M_{v,l}$
Death of mated female	$F_{w\times v,l} - 1$	$\mu_F F_{w\times v,l}$
Emigration of male	$M_{w,i} - 1$	$\xi_i^M M_{w,i}$
Emigration of unmated female	$F_w - 1$	$\xi_0^F F_w$
Emigration of mated female	$F_{w\times v,l} - 1$	$\xi_l^F F_{w\times v,l}$
Immigration of male	$M_{w,i} + 1$	ζ_i^M
Immigration of unmated female	$F_w + 1$	ζ_0^F
Immigration of mated female	$F_{w\times v,l} + 1$	ζ_l^F

2 Model generalisation

A generalised version for J strains of *Wolbachia* is depicted in Figure S1. The population progression is from "immature" ($I_{w,i} \forall w \in [1, \ldots, J]$ and $i \in [1, k]$) to adult male ($M_{w,j}$) or adult females that are unmated (F_w) or mated with males with strain v when the males were age l ($F_{w\times v,l}$).

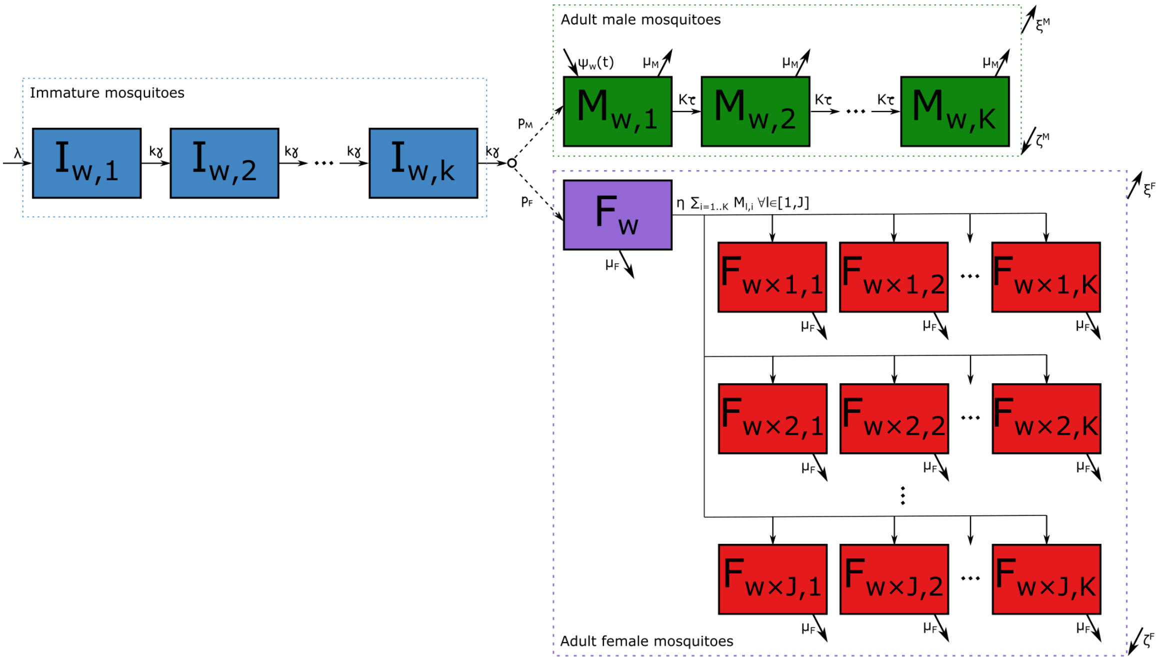

Figure S1: Schematic of the generalised mosquito population model for J strains of *Wolbachia*, where this depicts the progression for a single strain w of the Immature stages (blue) of strain w through to different male ages (green) or females who are unmated (purple) then mated with males with a specific *Wolbachia* strain and age (red). This is then repeated for the other $J-1$ strains. Immigration and emigration occurs from each adult compartment, captured here by the flows from the dashed boxes grouping compartments.

3 Wild-type steady states

To explore the intervention scenarios, we assume that the wild-type mosquito (wAlbAB) population is at an equilibrium before the introduction of any new species. Here, as done by Pagendam *et al.* [7], we determine the equilibrium states for the wild-type species with no invasive species added based on assumed observable mosquito population values. In §3.1 we assume a closed population model with no immigration or emigration, which is then added in §3.2. We determine biologically feasible parameter constraints in §3.3.

3.1 Closed population steady states

We apply an ordinary differential equation (ODE) approximation of the CTMC since we are studying a steady state. The following ODEs describe the dynamics, where dependence on *Wolbachia* strain has been dropped for simplicity of notation and $\dot{X}$ denotes the derivative of compartment X with respect to time:

$$\dot{I}_1 = \tilde{\lambda}\bar{F}_M - k\gamma I_1 \tag{S1}$$
$$\dot{I}_i = k\gamma I_{i-1} - k\gamma I_i\,,\; i = 2, 3, \ldots, k \tag{S2}$$
$$\dot{M}_1 = k\gamma p_m I_k - (\mu_M + K\sigma)M_1 \tag{S3}$$
$$\dot{M}_j = K\sigma M_{j-1} - (\mu_M + K\sigma)M_j\,,\; j = 2, 3, \ldots, K-1 \tag{S4}$$
$$\dot{M}_K = K\sigma M_{k-1} - \mu_M M_K \tag{S5}$$
$$\dot{F} = k\gamma p_f I_k - (\mu_F + \tilde{\eta})F \tag{S6}$$
$$\dot{F}_{M,l} = \eta M_l F - \mu_F F_{M,l}\,,\; l = 1, 2, \ldots, K, \tag{S7}$$

where

- $F_{M,l}$ is the mated female class with males of class l,
- $\tilde{\eta}$ is the basic mating rate given by $\tilde{\eta} = \eta\bar{M}$, $\bar{M} = \sum_{j=1}^{K} M_j$, and
- $\tilde{\lambda}$ is the density-dependant birth rate given by

  $$\tilde{\lambda} = \lambda\frac{(I_{max} - I_{total})}{I_{max}}\,,$$

  with I_{max} being the carrying capacity of larvae and $I_{total} = \sum_{i=1}^{k}(I_i)$.
- σ relates to age-related loss of cytoplasmic incompatibility by male mosquitoes, designed such that $K\sigma = 1$ for our scenarios.
- p_f and p_m are the probabilities an immature mosquito matures into an adult male or female, which must add to one. For the strains we study, $p_f = p_m = 1/2$.

Suppose we are given the maturation rate γ, birth rate λ, death rates μ_M and μ_F, the proportion of mated female mosquitoes at equilibrium p_{mated}, and equilibrium capacity of the adult population C. Then, following Pagendam *et al.* [7], we can calculate all equilibrium states and unknown

parameters of the System (S1)–(S7). In the usual way, setting the rates of change (left hand sides) of Equations (S1) and (S2) to zero gives steady state solutions denoted with a hat:

$$k\gamma\hat{I}_1 = \tilde{\lambda}\bar{F}_M$$

and

$$\hat{I}_i = \hat{I}_1$$

for all $i = 1, 2, \ldots, k$. This implies that

$$\hat{I}_i = \bar{I}$$

for a constant $\bar{I}$ independent of i. Equations (S3)–(S5) give

$$\begin{aligned}
\bar{I} &= \frac{\mu_M + K\sigma}{k\gamma p_m}\hat{M}_1\,, \\
\hat{M}_j &= \frac{K\sigma}{(\mu_M + K\sigma)}\hat{M}_{j-1}\,, \text{ for } j = 2, 3, \ldots, K-1, \text{ and,} \\
\hat{M}_K &= \frac{K\sigma}{\mu_M}\hat{M}_{K-1}\,.
\end{aligned}$$

Writing $\varphi = \frac{K\sigma}{\mu_M + K\sigma}$ gives

$$\bar{I} = \frac{K\sigma}{\varphi k\gamma p_m}\hat{M}_1\,, \tag{S8}$$

$$\hat{M}_j = \varphi^{j-1}\hat{M}_1\,, \text{ for } j = 1, 2, 3, \ldots, K-1, \text{ and,} \tag{S9}$$

$$\hat{M}_K = \frac{K\sigma\varphi^{K-2}}{\mu_M}\hat{M}_1\,. \tag{S10}$$

Hence, once we know $\hat{M}_1$ we know all immature states and all male states. To calculate $\hat{M}_1$, we first consider the total adult population at equilibrium

$$\bar{M} + \hat{F} + \bar{F}_M = C\,, \tag{S11}$$

where

$$\bar{M} = \sum_{j=1}^{K}\hat{M}_j$$

and

$$\bar{F}_M = \sum_{l=1}^{K}\hat{F}_{M,l}\,.$$

Noting that, at equilibrium, for the adult populations to remain constant the total deaths must equal the new maturations, such that

$$\begin{aligned}
\mu_M\bar{M} &= k\gamma p_m\hat{I}_k\,, \text{ and} \\
\mu_F\left(\hat{F} + \bar{F}_M\right) &= k\gamma p_f\hat{I}_k\,,
\end{aligned}$$

which means

$$\frac{\mu_M\bar{M}}{p_m} = \frac{\mu_F(\hat{F} + \bar{F}_M)}{p_f}\,.$$

This gives that the ratio

$$\frac{\hat{F}+\bar{F}_M}{\bar{M}} = \frac{\mu_M p_f}{\mu_F p_m} = \theta\,. \tag{S12}$$

Combining Equations (S11) and (S12) gives that

$$\bar{M} = \frac{C}{1+\theta}\,, \tag{S13}$$

$$\hat{F} = \frac{\theta}{1+\theta}(1-p_{mated})C\,, \text{ and} \tag{S14}$$

$$\bar{F}_M = \frac{\theta}{1+\theta} p_{mated} C\,, \tag{S15}$$

where $p_{mated} = \bar{F}_M/(\hat{F}+\bar{F}_M)$. Recalling that $\bar{M} = \sum_{j=1}^{K} \hat{M}_i$, then

$$\begin{aligned}\frac{C}{1+\theta} &= \bar{M} \\ &= \left[\sum_{j=1}^{K-1} \varphi^{j-1} + \frac{K\sigma\varphi^{K-2}}{\mu_M}\right]\hat{M}_1 \\ &= \left[\frac{1-\varphi^{K-1}}{1-\varphi} + \frac{K\sigma\varphi^{K-2}}{\mu_M}\right]\hat{M}_1\,.\end{aligned}$$

Hence

$$\hat{M}_1 = \frac{C}{(1+\theta)\left[\frac{1-\varphi^{K-1}}{1-\varphi} + \frac{K\sigma\varphi^{K-2}}{\mu_M}\right]}\,. \tag{S16}$$

Now only two quantities remain unknown: the larvae population limits I_{max} and the mating rate η. To find I_{max}, we equate Equation (S1) to zero giving

$$\begin{aligned}\bar{I} &= \frac{\tilde{\lambda}}{k\gamma}\hat{F}_M \\ &= \frac{\hat{F}_M \lambda}{k\gamma}\frac{\left(I_{max} - \hat{I}_{total}\right)}{I_{max}}\,,\end{aligned}$$

where $\hat{I}_{total} = \sum_{i=1}^{k} \hat{I}_i = k\bar{I}$. Rearranging for I_{max} then gives

$$I_{max} = \frac{k\bar{I}}{1 - \dfrac{k\gamma\bar{I}}{\lambda\bar{F}_M}}\,. \tag{S17}$$

Finally, to calculate the mating rate η we set Equation (S6) to zero to get

$$k\gamma p_f \bar{I} = \mu_F \hat{F} + \eta\bar{M}\hat{F}$$

and so

$$\eta = \frac{k\gamma p_f \bar{I} - \mu_F \hat{F}}{\bar{M}\hat{F}}\,. \tag{S18}$$

From Equation (S7),

$$\hat{F}_{M,l} = \frac{\eta \hat{M}_l \hat{F}}{\mu_F}\,, \tag{S19}$$

and so all steady state values can be determined.

3.2 Immigration and emigration

We introduce immigration and emigration into the system such that the steady states found in Section 3 remain unchanged. Assuming no immigration at the immature stages and denoting immigration by ζ^s and emigration by ξ^s ($s \in \{M, F\}$), Equations (S1)-(S7) become:

$$\dot{I}_1 = \tilde{\lambda} \bar{F}_M - k\gamma I_1 \tag{S20}$$
$$\dot{I}_i = k\gamma I_{i-1} - k\gamma I_i\,,\; i = 2, 3, \ldots, k \tag{S21}$$
$$\dot{M}_1 = k\gamma p_m I_{m,k} - (\mu_M + K\sigma) M_1 + \zeta_1^M - \xi_1^M M_1 \tag{S22}$$
$$\dot{M}_j = K\sigma M_{j-1} - (\mu_M + K\sigma) M_j + \zeta_j^M - \xi_j^M M_j\,,\; j = 2, 3, \ldots, K-1 \tag{S23}$$
$$\dot{M}_K = K\sigma M_{k-1} - \mu_M M_K + \zeta_K^M - \xi_K^M M_K \tag{S24}$$
$$\dot{F} = k\gamma p_f I_{f,k} - (\mu_F + \tilde{\eta}) F + \zeta_0^F - \xi_0^F F \tag{S25}$$
$$\dot{F}_{M,l} = \eta M_l F - \mu_F F_{M,l} + \zeta_l^F - \xi_l^F F_{M,l}\,,\; l = 1, 2, \ldots, K, \tag{S26}$$

To maintain the steady states of Section 3, we require at equilibrium that

$$\zeta_j^M = \xi_j^M \hat{M}_j\,, j = 1, 2, \ldots, K, \text{ and} \tag{S27}$$
$$\zeta_l^F = \xi_l^M \hat{F}_{M,l}\,, l = 0, 1, 2, \ldots, K, \tag{S28}$$

where $\hat{F}_{M,0} = \hat{F}$. We first focus on the immigration rate. We assume the following:

A1 There are overall immigration per capita rates for males and females.

A2 Immigration is from an external patch with equal population size which is already in an equilibrium state.

A3 The rate of immigration depends on mosquito age, not mate status.

Looking at the male immigration rate, Assumption A1 tells us that there is a constant ζ^M such that

$$\sum_{j=1}^{K} \zeta_j^M = \zeta^M\,,$$

whereas Assumption A2 says

$$\zeta_j^M = \tilde{\zeta}_j^M \hat{M}_j\,,$$

that is, ζ_j^M is proportional to the number of male mosquitoes aged j in the steady state solutions. Combining these gives

$$\begin{aligned}\zeta^M &= \sum_{j=1}^{K} \zeta_j^M \\ &= \sum_{j=1}^{K} \tilde{\zeta}_j^M \hat{M}_j \\ &= \left(\sum_{j=1}^{K-1} \left(\tilde{\zeta}_j^M \varphi^{j-1} \right) + \frac{K\sigma\varphi^{K-2}}{\mu_M} \tilde{\zeta}_K^M \right) \hat{M}_1 \,,\end{aligned}$$

which gives

$$\left(\sum_{j=1}^{K-1} \left(\tilde{\zeta}_j^M \varphi^{j-1} \right) + \frac{K\sigma\varphi^{K-2}}{\mu_M} \tilde{\zeta}_K^M \right) = \frac{\zeta^M}{\hat{M}_1} \,. \tag{S29}$$

Now, even with the constraint that $\tilde{\zeta}_j^M \geq 0$ for all $j = 1, 2, \ldots, K$, Equation (S29) defines a whole surface of solutions. Thus, we constrain the problem with Assumption A3 and assume that

$$\tilde{\zeta}_j^M = \tilde{\zeta}^M P_j \,, \tag{S30}$$

where $\tilde{\zeta}^M$ is constant and P_j is the probability that a male mosquito lives to be age j. Let Y denote the time a male mosquito is alive, which from adding Equations (S3)–(S5) we find is exponentially distributed with rate μ_M. Then

$$\begin{aligned}P_j &= P(m \in M_j) \\ &= P\left(\frac{j-1}{K\sigma} < Y \leq \frac{j}{K\sigma} \right) \\ &= e^{-\mu_M (j-1)/(K\sigma)} - e^{-\mu_M j/(K\sigma)} \,.\end{aligned}$$

Note we have divided the index j by the transition between male age classes $K\sigma$. Since $j \leq K$, when $j = K$ we get

$$P_K = P\left(Y > \frac{j-1}{K\sigma} \right) \,.$$

Incorporating Equation (S30) into (S29) gives that

$$\tilde{\zeta}^M = \frac{\zeta^M}{\hat{M}_1 \left(\sum_{j=1}^{K-1} \left(P_j \varphi^{j-1} \right) + \frac{K\sigma\varphi^{K-2}}{\mu_M} P_K \right)} \,. \tag{S31}$$

Using Equation (S27) gives that the emigration rate is

$$\xi_j^M = \tilde{\zeta}_j^M$$

for all $j = 1, 2, \ldots, K$. Now consider the female immigration rate. Again, Assumptions A1 and A2 give us that

$$\sum_{l=0}^{K} \tilde{\zeta}_l^F \hat{F}_{M,l} = \zeta^F \,,$$

but now Assumption A3 tells us that this only depends on mosquito age and not mating status. Since we only consider one age strata for female mosquitoes, Assumption A3 implies that there is a constant $\tilde{\zeta}^F$ such that

$$\tilde{\zeta}_l^F = \tilde{\zeta}^F$$

for all $l = 0, 1, 2, \ldots, K$. Thus,

$$\tilde{\zeta}^F = \frac{\zeta^F}{\sum_{l=0}^{K} \hat{F}_{M,l}} .$$

Noting that

$$\begin{aligned}\sum_{l=0}^{K} \hat{F}_{M,l} &= \hat{F} + \bar{F}_M \\ &= \frac{\theta}{1+\theta} C ,\end{aligned}$$

we get

$$\tilde{\zeta}^F = \frac{(1+\theta)\zeta^F}{\theta C} . \tag{S32}$$

Again, Equation (S27) gives that the emigration rate is

$$\xi_l^F = \tilde{\zeta}^F$$

for all $l = 0, 1, 2, \ldots, K$. In summary and for clarity, we get that

$$\begin{aligned}\zeta_j^M &= \frac{\zeta^M P_j \hat{M}_j}{\hat{M}_1 \left(\sum_{i=1}^{K-1} (P_i \varphi^{i-1}) + \dfrac{K\sigma\varphi^{K-2}}{\mu_M} P_K \right)} , \\ \xi_j^M &= \frac{\zeta^M P_j}{\hat{M}_1 \left(\sum_{i=1}^{K-1} (P_i \varphi^{i-1}) + \dfrac{K\sigma\varphi^{K-2}}{\mu_M} P_K \right)} , \\ \zeta_l^F &= \frac{(1+\theta)\zeta^F}{\theta C} \hat{F}_{M,l}, \\ \xi_l^F &= \frac{(1+\theta)\zeta^F}{\theta C} ,\end{aligned}$$

for $j = 1, 2, \ldots, K$ and $l = 0, 1, 2, \ldots, K$.

3.3 Biologically feasible constraints on parameters

The calculation of the secondary parameters in Section 3 gives a constraint on the parameters for the model to give biologically feasible results. Equation (S17) gives that

$$\frac{k\gamma\bar{I}}{\lambda\bar{F}_M} < 1 .$$

First considering the numerator, substituting $\bar{I}$ from (S8) gives

$$\begin{aligned}k\gamma\bar{I} &= k\gamma \frac{K\sigma}{\varphi k \gamma p_m} \hat{M}_1 \\ &= \frac{K\sigma C}{p_m (1+\theta) \varphi \left[\frac{1-\varphi^{K-1}}{1-\varphi} + \frac{K\sigma\varphi^{K-2}}{\mu_M} \right]} .\end{aligned}$$

Noting that $1-\varphi = (\mu_M\varphi)/(K\sigma)$, this reduces to

$$k\gamma\bar{I} = \frac{C\mu_M}{p_m(1+\theta)}\,. \tag{S33}$$

Considering the denominator, substitute in Equation (S15),

$$\lambda\bar{F}_M = \lambda p_{mated}C\frac{\theta}{1+\theta}\,.$$

Hence,

$$\begin{aligned}\frac{k\gamma\bar{I}}{\lambda\bar{F}_M} &= \frac{\frac{C\mu_M}{p_m(1+\theta)}}{\lambda p_{mated}C\frac{\theta}{1+\theta}}\\ &= \frac{\mu_M}{p_m\lambda p_{mated}\theta}.\end{aligned}$$

Recalling that $\theta = \frac{\mu_M p_f}{\mu_F p_m}$ gives the constraint

$$\frac{\mu_F}{p_f\lambda p_{mated}} < 1\,. \tag{S34}$$

This effectively just states that female mosquitoes must be born (noting immatures all become adults) at a greater rate than they die. The strict inequality is likely a consequence of the density-dependence (i.e. due to $\tilde{\lambda}$).

Note that there seems to be a second constraint from Equation (S18), which requires that

$$\frac{k\gamma p_f\bar{I}}{\mu_F\hat{F}} > 1\,.$$

However, combining Equations (S33), (S14), and (S12) gives that

$$\frac{k\gamma p_f\bar{I}}{\mu_F\hat{F}} = \frac{1}{1-p_{mated}}\,.$$

Hence, this constraint is met for all biologically feasible $p_{mated} \in (0,1)$.

3.4 Multiple strains of *Wolbachia*

When considering $J \geq 2$ strains of *Wolbachia* we modify the stoichiometries of Table S2 in the following ways. First, all immature mosquitoes, regardless of *Wolbachia* strain, contribute to the total immature pool I_{total}. This feature creates a resource competition between the multiple strains of *Wolbachia*. Second, the mating rate η (Equation S18) between a female of strain w and a male of age l strain v is modified by Fried's index and mating competitiveness. Mating pressure for type v and age l is defined as

$$\kappa_{v,l} = \text{Fried}_v M_{v,l}.$$

Then the mating rate is modified by the mating competitiveness between age and strain as

$$\eta_{v,l} = \eta\frac{\kappa_{v,l}}{\sum_{v,l}\kappa_{v,l}}\,.$$

This modification means that mosquito mating competitiveness is proportional to their density in the population. The final modification is through the birthing rate. When mosquitoes of two different strains w and v of *Wolbachia* mate there is an effect of CI that reduces offspring viability. This is represented in our model as modifying the birth rate $\tilde{\lambda}$ as

$$\tilde{\lambda}_{w\times v,l} = (1 - c_{w\times v,l})\tilde{\lambda}\,,$$

where $c_{w\times v,l}$ is the CI between *Wolbachia* strains w and v for an adult male of age l.

4 Sensitivity to Fried's index

We assess the qualitative sensitivity of our results to Fried's index by running 500 simulations using the expected parameters of Table S1 and varying Fried's index. We run these simulations using $\text{Fried}_{ARwP} \in \{0.36, 0.73\}$, which are suggested values for ARwP competitiveness in recent field trials [4].

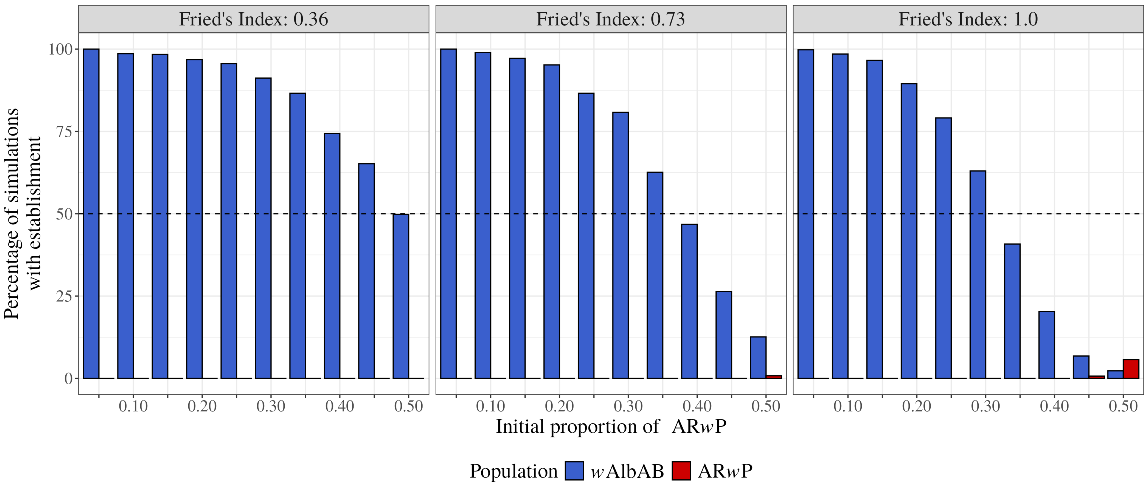

Figure S2: Percentage of simulations with establishment of each population at the end of the 500 *in silico* cage simulations for different values of Fried's index. A population is established if the proportion of adults is greater than 10% of the initial population size. The left panel shows simulations with Fried's index = 0.36, the middle panel shows simulations with Fried's index = 0.73, and the right panel shows simulations with Fried's index = 1.00. Red columns represent the ARwP population and blue columns the wAlbAB population. The x-axis indicates the initial proportion of ARwP, and the y-axis shows the percentage of simulations that resulted in establishment. The dotted horizontal line marks 50% of simulations. Each simulation started with 420 adult mosquitoes (half male, half female), and the fitness parameters are the expected values defined in Table S1. Only bi-directional cytoplasmic incompatibility was considered in these simulations.

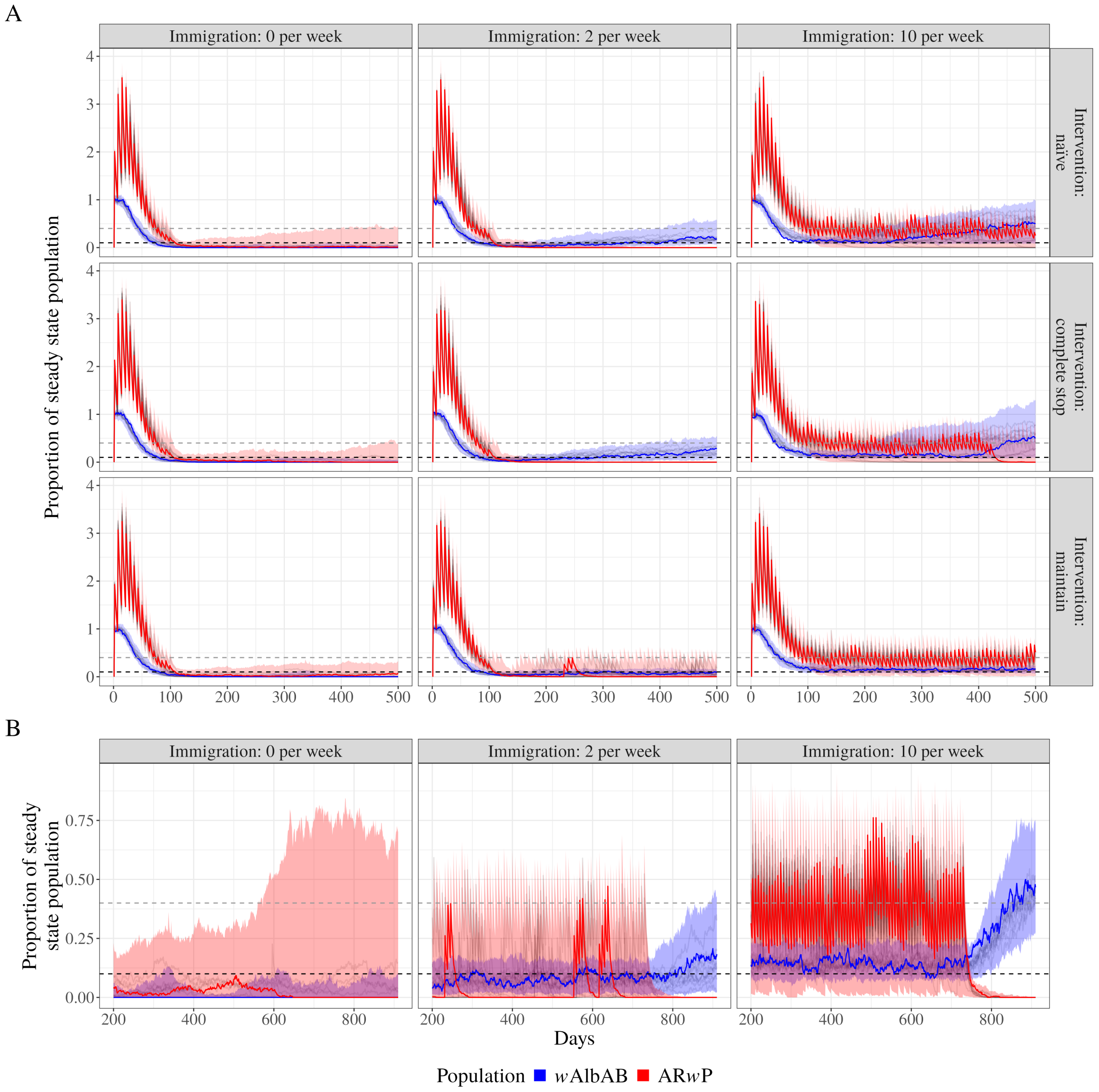

Figure S3: Proportion of mosquitoes relative to the steady-state population over time for different release strategies (rows) and immigration rates (columns) with Fried's index = 0.36. The ARwP population (red) and wAlbAB population (blue) lines represent the median with 95% confidence intervals (shaded areas). Black lines represent 10 random simulation runs. Panel A displays the population over 500 days under three immigration rates (0, 2, and 10 mosquitoes per week). The intervention strategies include naïve, complete, and maintain. Panel B focuses on the maintain intervention strategy over days 200 to 920 for the same immigration rates. Horizontal dotted lines represent the suppression/establishment (10%) and the unstable equilibrium ω^* (40%) thresholds used. Values above 1 on the y-axis indicate population levels higher than the initial steady state. Other model parameters are the expected values defined in Table S1.

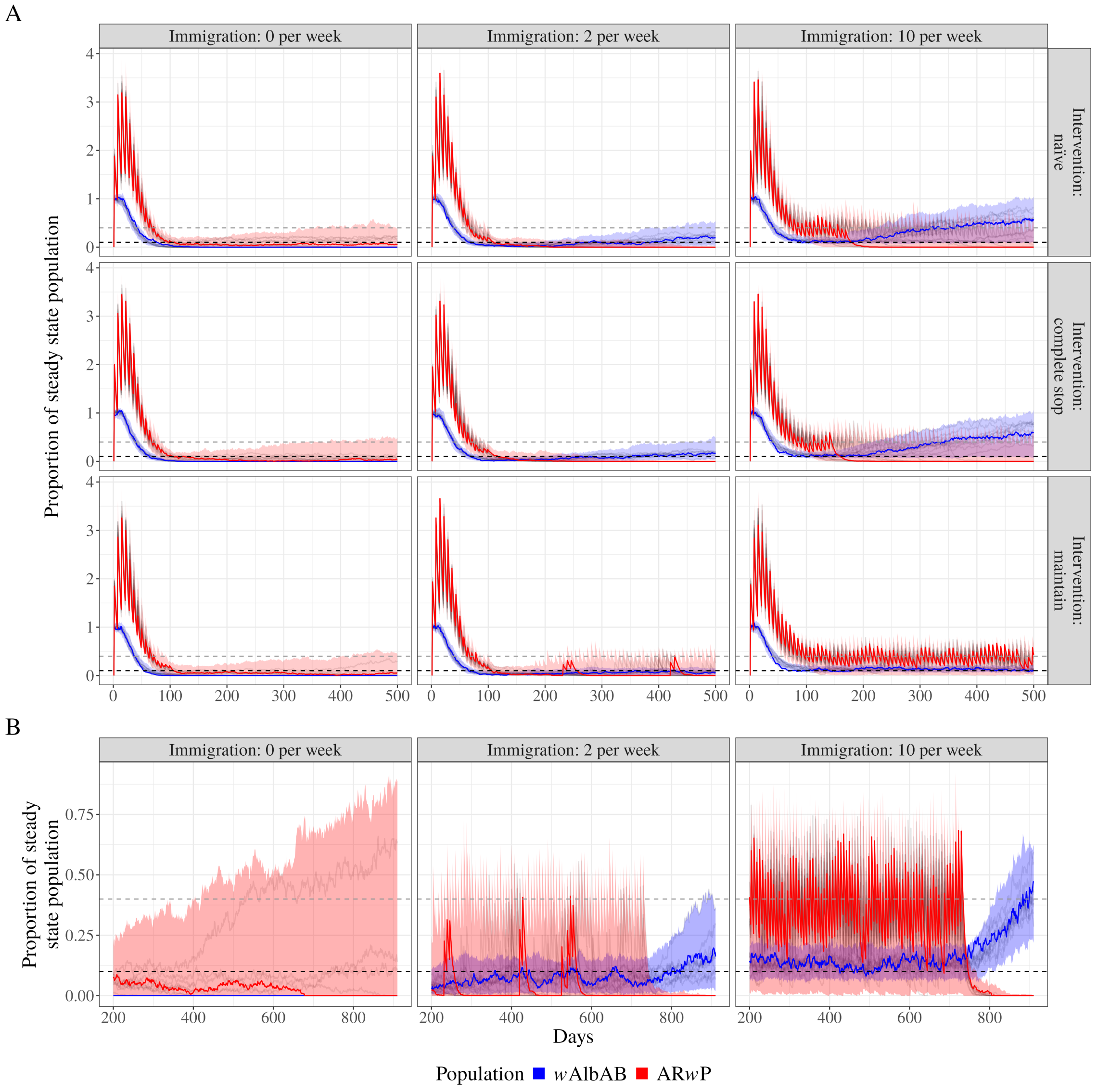

Figure S4: Proportion of mosquitoes relative to the steady-state population over time for different release strategies (rows) and immigration rates (columns) with Fried's index = 0.73. The ARwP population (red) and wAlbAB population (blue) lines represent the median with 95% confidence intervals (shaded areas). Black lines represent 10 random simulation runs. Panel A displays the population over 500 days under three immigration rates (0, 2, and 10 mosquitoes per week). The intervention strategies include naïve, complete, and maintain. Panel B focuses on the maintain intervention strategy over days 200 to 920 for the same immigration rates. Horizontal dotted lines represent the suppression/establishment (10%) and the unstable equilibrium ω^* (40%) thresholds used. Values above 1 on the y-axis indicate population levels higher than the initial steady state. Other model parameters are the expected values defined in Table S1.

Table S3: Percentage of simulations with management success for wAlbAB and ARwP populations under different scenarios for immigration rates and intervention strategies and varying values of Fried's index. Management success is defined as keeping the wAlbAB population below 10% of the initial population size, and ARwP below 40% (unstable equilibrium threshold). The data is shown both within the stopping time and six months after. **The other model parameters are the expected values defined in Table S1.**

Scenario (Fried's index = 0.36)	Naïve		Complete stop		Maintain	
	wAlbAB	ARwP	wAlbAB	ARwP	wAlbAB	ARwP
**0 per week**						
Within stopping time	100	100	100	100	100	100
After six months	100	100	100	100	100	100
**2 per week**						
Within stopping time	100	100	100	100	89.6	100
After six months	50	100	48.6	100	35.2	100
**10 per week**						
Within stopping time	85.6	99.8	81.2	96.6	8.6	100
After six months	0	100	0	100	0	100
Scenario (Fried's index = 0.73)	Naïve		Complete stop		Maintain	
	wAlbAB	ARwP	wAlbAB	ARwP	wAlbAB	ARwP
**0 per week**						
Within stopping time	100	100	100	100	100	100
After six months	100	100	100	100	100	100
**2 per week**						
Within stopping time	100	100	100	100	92.8	100
After six months	73.2	100	76	100	49.7	100
**10 per week**						
Within stopping time	99.4	100	98.8	99.6	16.4	100
After six months	0	100	0	100	0	100

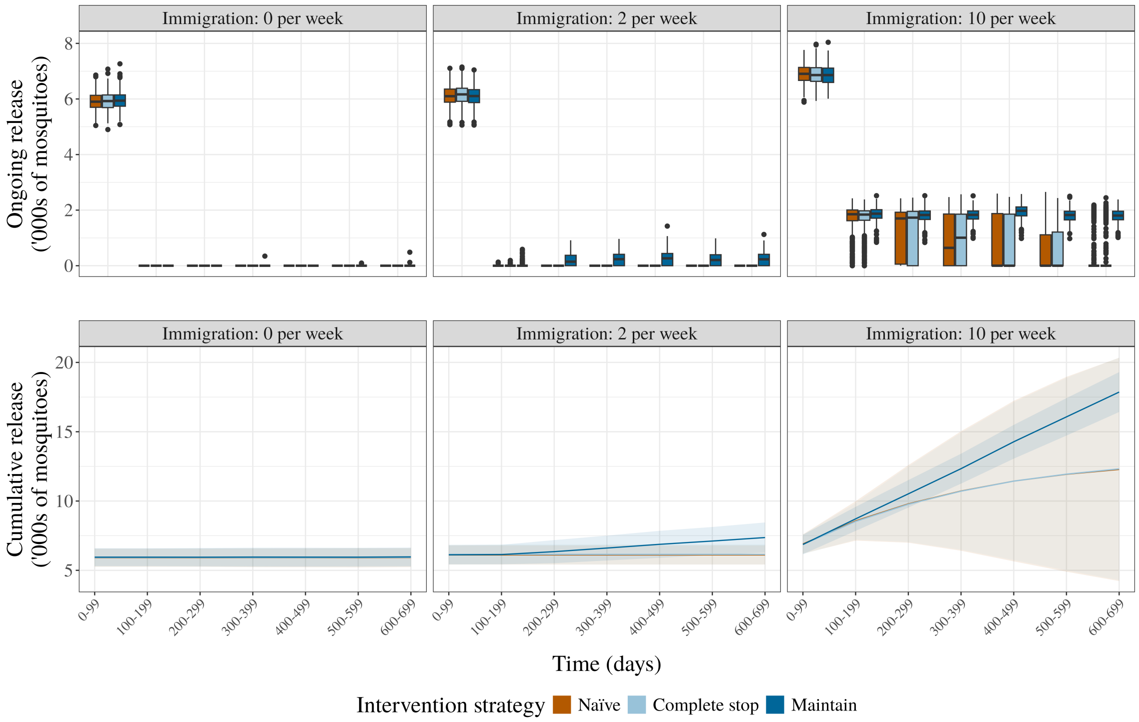

Figure S5: The release in thousands of ARwP mosquitoes as a proxy for cost under different intervention strategies and immigration rates with Fried's index = 0.36. The x-axis represents the time in 100-day intervals up until day 700. The top row shows the ongoing cost every hundred days, measured as the number of mosquitoes released every 100 days for each intervention strategy (naïve, complete stop, and maintain). The bottom row illustrates the cumulative release (in thousands of mosquitoes) over the entire period for each strategy. The model parameters are the expected values defined in Table S1.

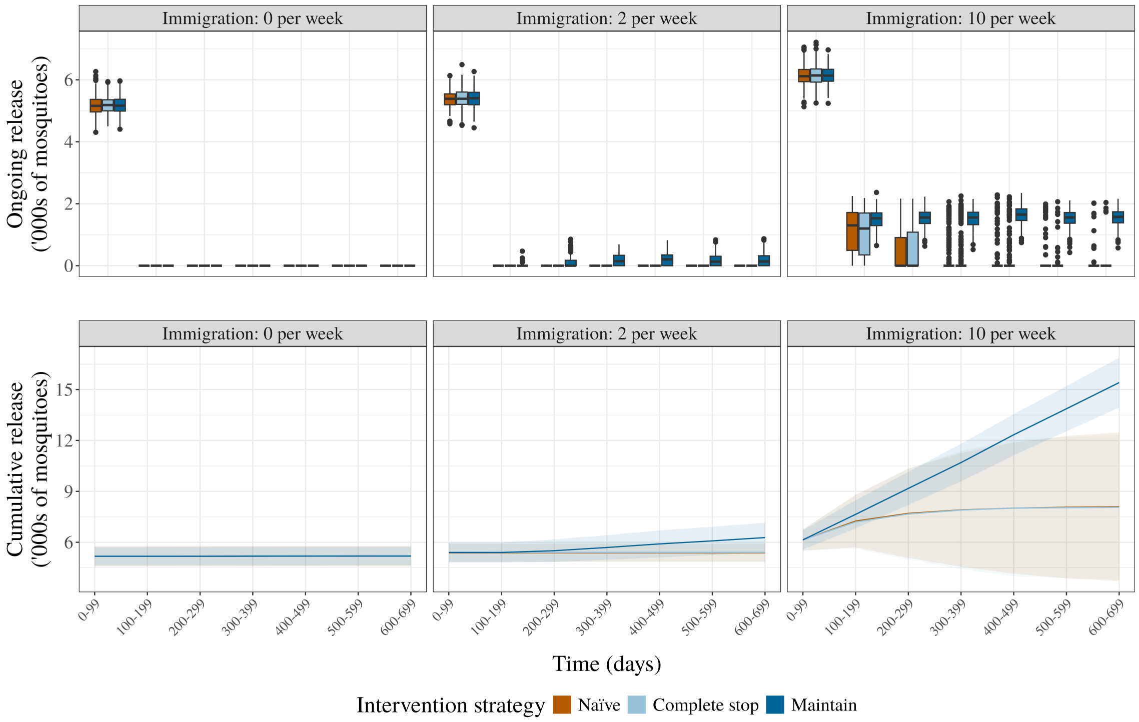

Figure S6: The release in thousands of ARwP mosquitoes as a proxy for cost under different intervention strategies and immigration rates with Fried's index = 0.73. The x-axis represents the time in 100-day intervals up until day 700. The top row shows the ongoing cost every hundred days, measured as the number of mosquitoes released every 100 days for each intervention strategy (naïve, complete stop, and maintain). The bottom row illustrates the cumulative release (in thousands of mosquitoes) over the entire period for each strategy. The model parameters are the expected values defined in Table S1.

5 Additional figures and tables

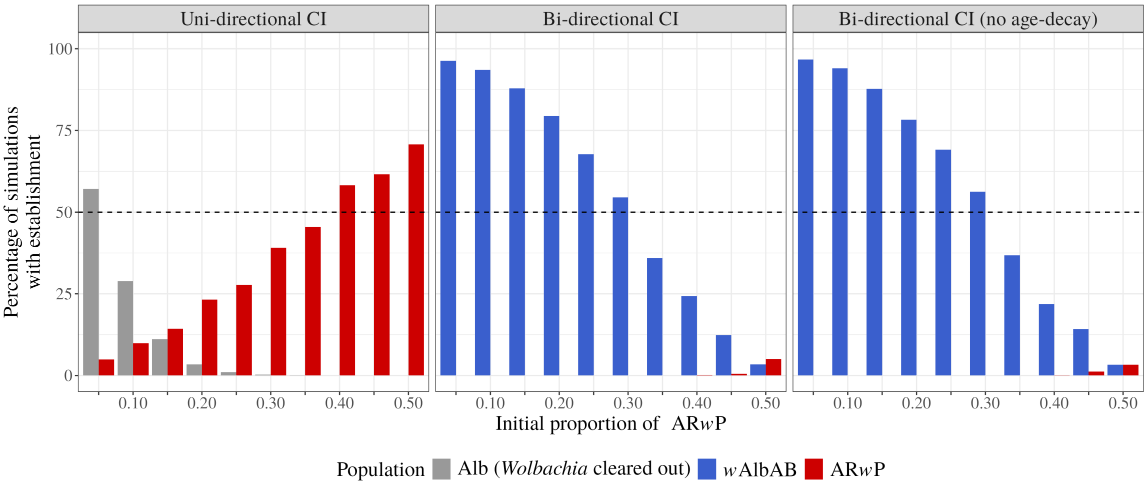

Figure S7: Percentage of simulations with establishment of each population at the end of the 500 *in silico* cage simulations. A population is established if the proportion of adults is greater than 10% of the initial population size. The left panel shows simulations with uni-directional Cytoplasmic Incompatibility (CI), the middle panel depicts bi-directional CI with age-decay and the right panel bi-directional CI without age-decay. Red columns represent the ARwP population, blue columns the wAlbAB population, and grey columns the *Wolbachia* cleared out wild-type population. The x-axis indicates the initial proportion of ARwP, and the y-axis shows the percentage of simulations that resulted in establishment. The dotted horizontal line marks 50% of simulations. Each simulation started with 420 adult mosquitoes (half male, half female), and the fitness parameters are the low values defined in Table S1.

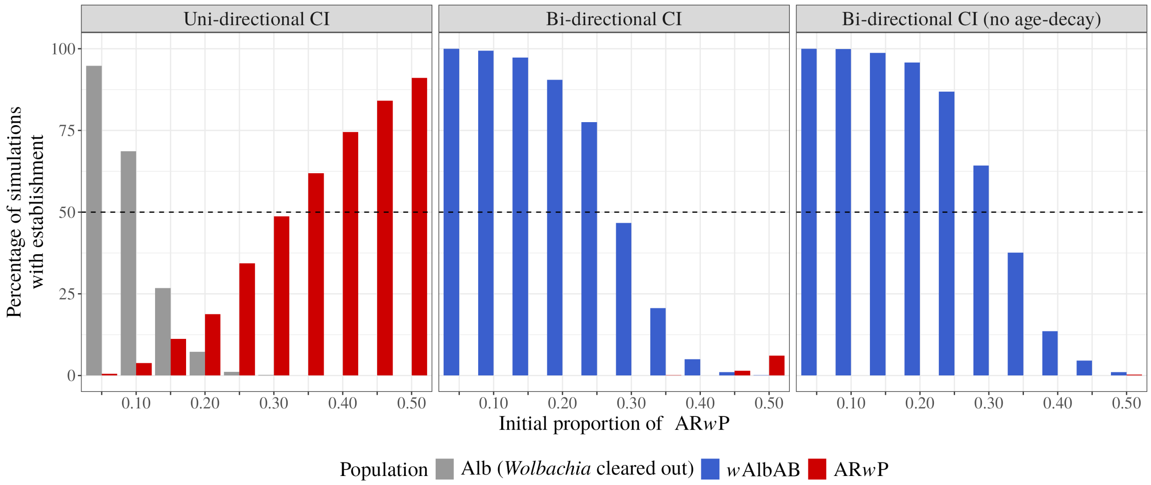

Figure S8: Percentage of simulations with establishment of each population at the end of the 500 *in silico* cage simulations. A population is established if the proportion of adults is greater than 10% of the initial population size. The left panel shows simulations with uni-directional Cytoplasmic Incompatibility (CI), the middle panel depicts bi-directional CI with age-decay and the right panel bi-directional CI without age-decay. Red columns represent the ARwP population, blue columns the wAlbAB population, and grey columns the *Wolbachia* cleared out wild-type population. The x-axis indicates the initial proportion of ARwP, and the y-axis shows the percentage of simulations that resulted in establishment. The dotted horizontal line marks 50% of simulations. Each simulation started with 420 adult mosquitoes (half male, half female), and the fitness parameters are the high values defined in Table S1.

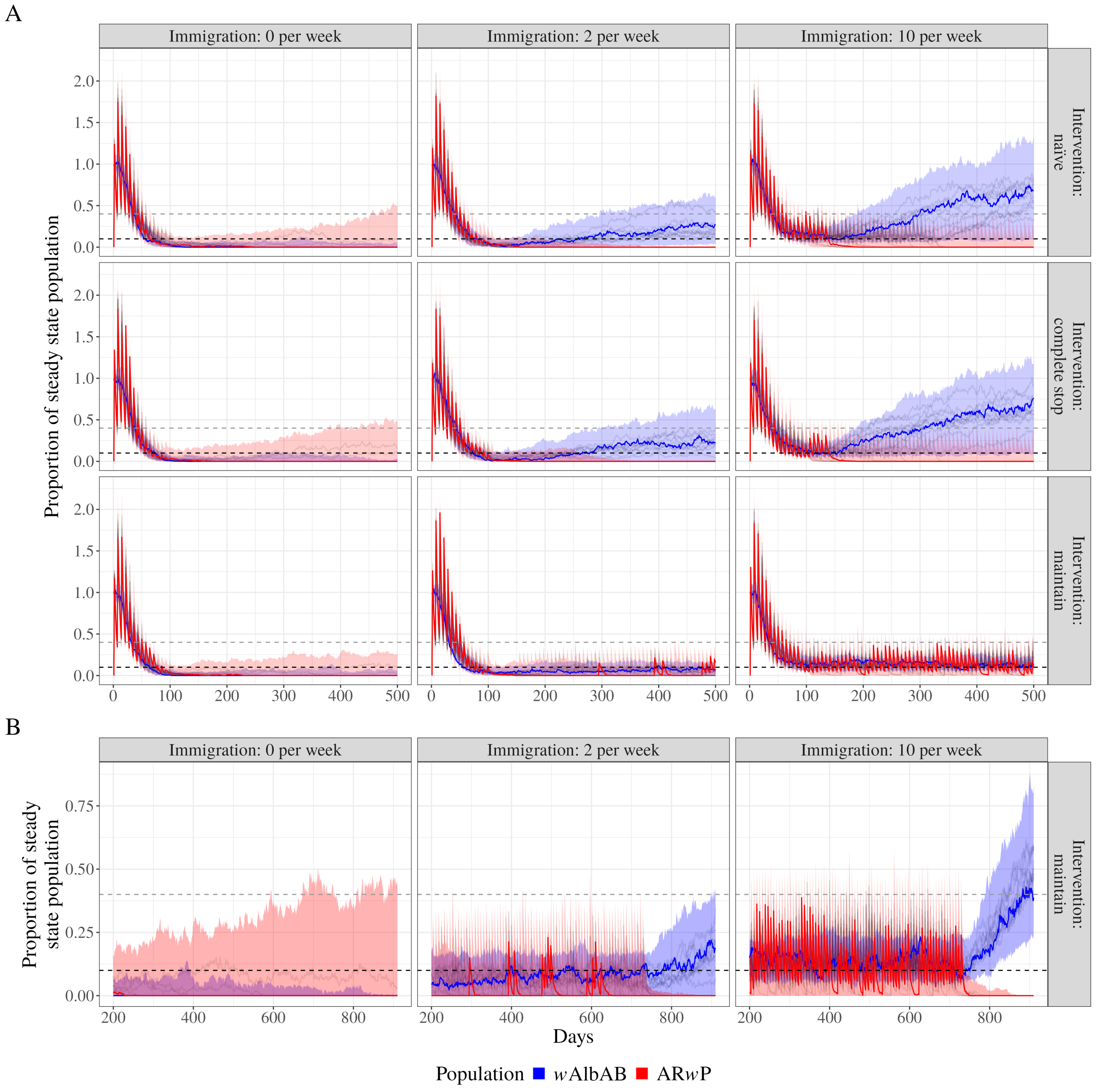

Figure S9: Proportion of mosquitoes relative to the steady-state population over time for different release strategies (rows) and immigration rates (columns). The ARwP population (red) and wAlbAB population (blue) lines represent the median with 95% confidence intervals (shaded areas). Black lines represent 10 random simulation runs. Panel A displays the population over 500 days under three immigration rates (0, 2, and 10 mosquitoes per week). The intervention strategies include naïve, complete, and maintain. Panel B focuses on the maintain intervention strategy over days 200 to 920 for the same immigration rates. Horizontal dotted lines represent the suppression/establishment (10%) and the unstable equilibrium ω^* (40%) thresholds used. Values above 1 on the y-axis indicate population levels higher than the initial steady state. Model parameters are the low values defined in Table S1.

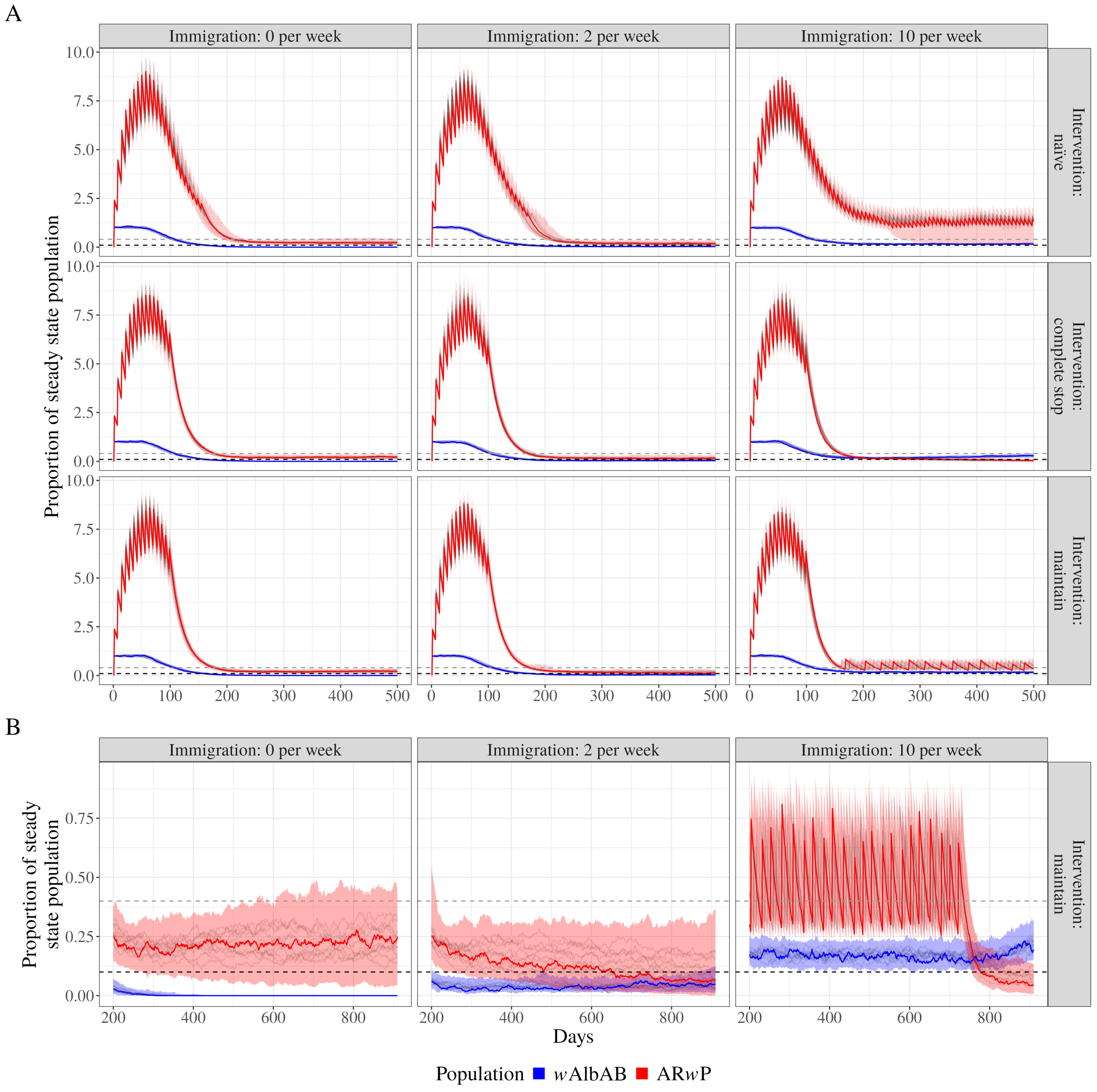

Figure S10: Proportion of mosquitoes relative to the steady-state population over time for different release strategies (rows) and immigration rates (columns). The ARwP population (red) and wAlbAB population (blue) lines represent the median with 95% confidence intervals (shaded areas). Black lines represent 10 random simulation runs. Panel A displays the population over 500 days under three immigration rates (0, 2, and 10 mosquitoes per week). The intervention strategies include naïve, complete, and maintain. Panel B focuses on the maintain intervention strategy over days 200 to 920 for the same immigration rates. Horizontal dotted lines represent the suppression/establishment (10%) and the unstable equilibrium ω^* (40%) thresholds used. Values above 1 on the y-axis indicate population levels higher than the initial steady state. Model parameters are the high values defined in Table S1.

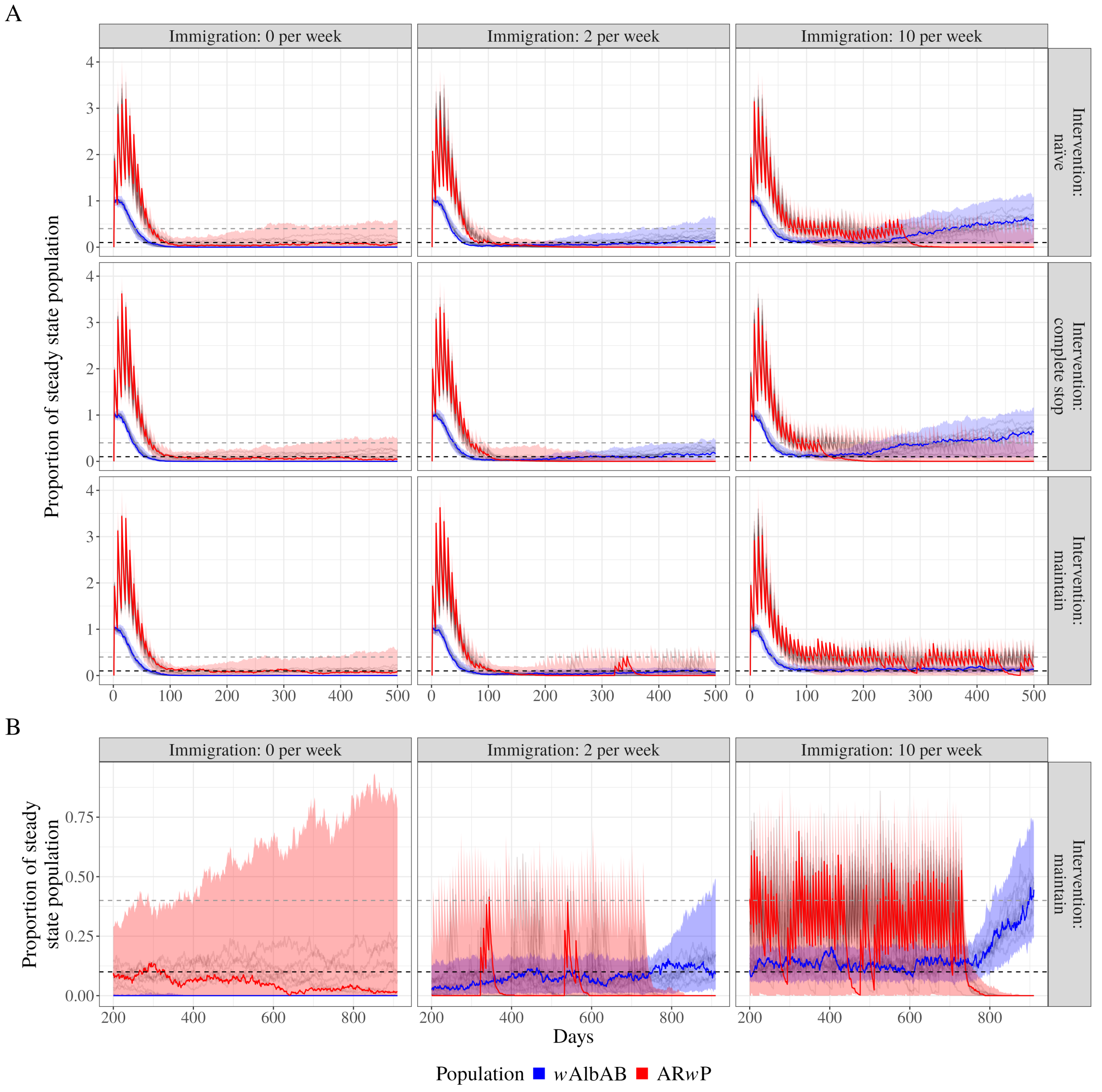

Figure S11: Proportion of mosquitoes relative to the steady-state population over time for different release scenarios (rows) and immigration rates (columns) and no age-related CI decay. The ARwP population (red) and wAlbAB population (blue) lines represent the median with 95% confidence intervals (shaded areas). Black lines represent 10 random simulation runs. Panel A displays the population over 500 days under three immigration rates (0, 2, and 10 mosquitoes per week). The intervention scenarios include naïve, complete, and maintain. Panel B focuses on the maintain scenario over days 200 to 920 for the same immigration rates. Horizontal dotted lines represent the suppression/establishment (10%) and the unstable equilibrium ω^* (40%) thresholds used. Values above 1 on the y-axis indicate population levels higher than the initial steady state. Model parameters are the expected values defined in Supplementary Materials Table S1.

Table S4: Percentage of simulations with management success for *w*AlbAB and AR*w*P populations under different scenarios for immigration rates and intervention strategies. Management success is defined as keeping the *w*AlbAB population below 10% of the initial population size, and AR*w*P below 40% (unstable equilibrium threshold). The data is shown both within the stopping time and six months after. The model parameters are the low values defined in Table S1.

Scenario	Naïve		Complete stop		Maintain	
	*w*AlbAB	AR*w*P	*w*AlbAB	AR*w*P	*w*AlbAB	AR*w*P
**0 per week**						
Within stopping time	100	100	100	100	100	100
After six months	100	100	100	100	100	100
**2 per week**						
Within stopping time	100	100	100	100	91.2	100
After six months	50.6	100	44.6	100	43.0	100
**10 per week**						
Within stopping time	100	100	100	100	19.2	100
After six months	0.0	100	0.0	100	0.0	100

Table S5: Percentage of simulations with management success for *w*AlbAB and AR*w*P populations under different scenarios for immigration rates and intervention strategies. Management success is defined as keeping the *w*AlbAB population below 10% of the initial population size, and AR*w*P below 40% (unstable equilibrium threshold). The data is shown both within the stopping time and six months after. The model parameters are the high values defined in Table S1.

Scenario	Naïve		Complete stop		Maintain	
	*w*AlbAB	AR*w*P	*w*AlbAB	AR*w*P	*w*AlbAB	AR*w*P
**0 per week**						
Within stopping time	100	0.0	0.0	0.0	0.0	0.0
After six months	100	100	100	100	100	100
**2 per week**						
Within stopping time	100	0.0	0.0	0.0	6.6	0.8
After six months	100	100	100	100	100	100
**10 per week**						
Within stopping time	0.6	0.0	0.0	0.0	0.0	1.0
After six months	0.0	100	0.0	100	0.0	100

Table S6: Percentage of simulations with management success for wAlbAB and ARwP populations under different scenarios for immigration rates and intervention strategies with no age-related CI decay. Management success is defined as keeping the wAlbAB population below 10% of the initial population size, and ARwP below 40% (unstable equilibrium threshold). The data is shown both within the stopping time and six months after. The model parameters are the expected values defined in Table S1.

Scenario	Naïve		Complete stop		Maintain	
	wAlbAB	ARwP	wAlbAB	ARwP	wAlbAB	ARwP
**0 per week**						
Within stopping time	100	100	100	100	100	100
After six months	100	99.9	100	99.9	100	99.9
**2 per week**						
Within stopping time	100	100	100	100	92.6	100
After six months	77.9	100	76.2	100	49.7	100
**10 per week**						
Within stopping time	99.9	100	99.6	99.7	23.8	100
After six months	0	100	0	100	0	100

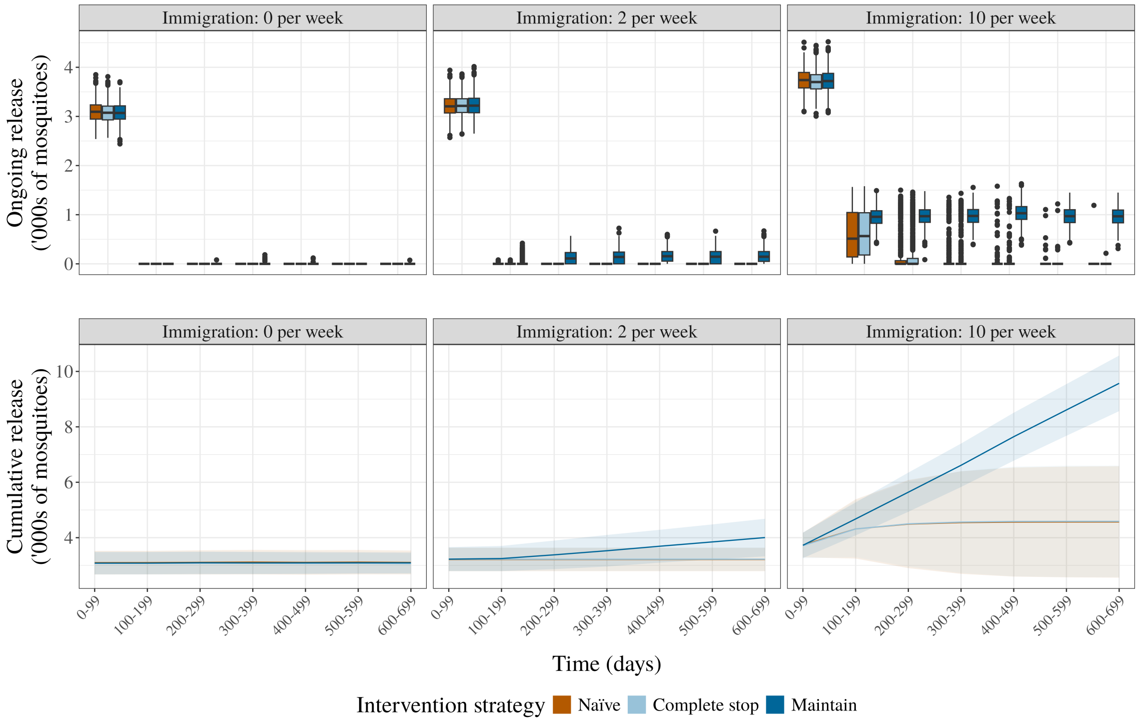

Figure S12: The release in thousands of ARwP mosquitoes as a proxy for cost under different intervention strategies and immigration rates. The x-axis represents the time in 100-day intervals up until day 700. The top row shows the ongoing cost every hundred days, measured as the number of mosquitoes released every 100 days for each intervention strategy (naïve, complete stop, and maintain). The bottom row illustrates the cumulative release (in thousands of mosquitoes) over the entire period for each strategy. The model parameters are the low values defined in Table S1.

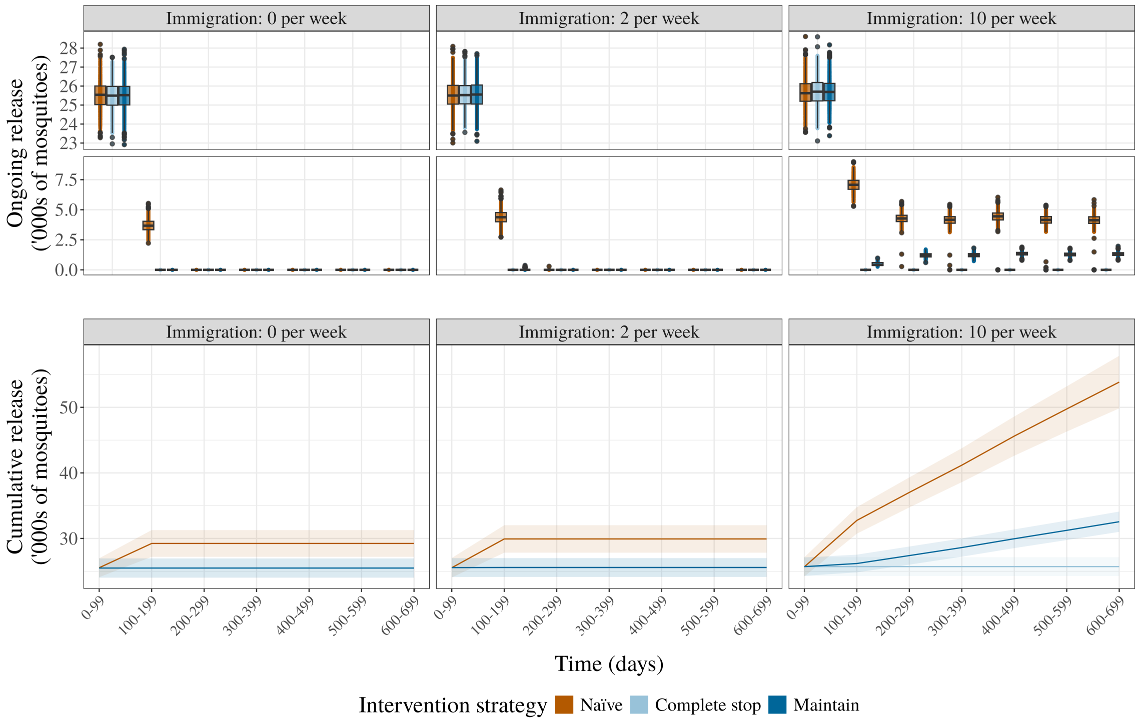

Figure S13: The release in thousands of ARwP mosquitoes as a proxy for cost under different intervention strategies and immigration rates. The x-axis represents the time in 100-day intervals up until day 700. The top row shows the ongoing cost every hundred days, measured as the number of mosquitoes released every 100 days for each intervention strategy (naïve, complete stop, and maintain). The bottom row illustrates the cumulative release (in thousands of mosquitoes) over the entire period for each strategy. The model parameters are the high values defined in Table S1.

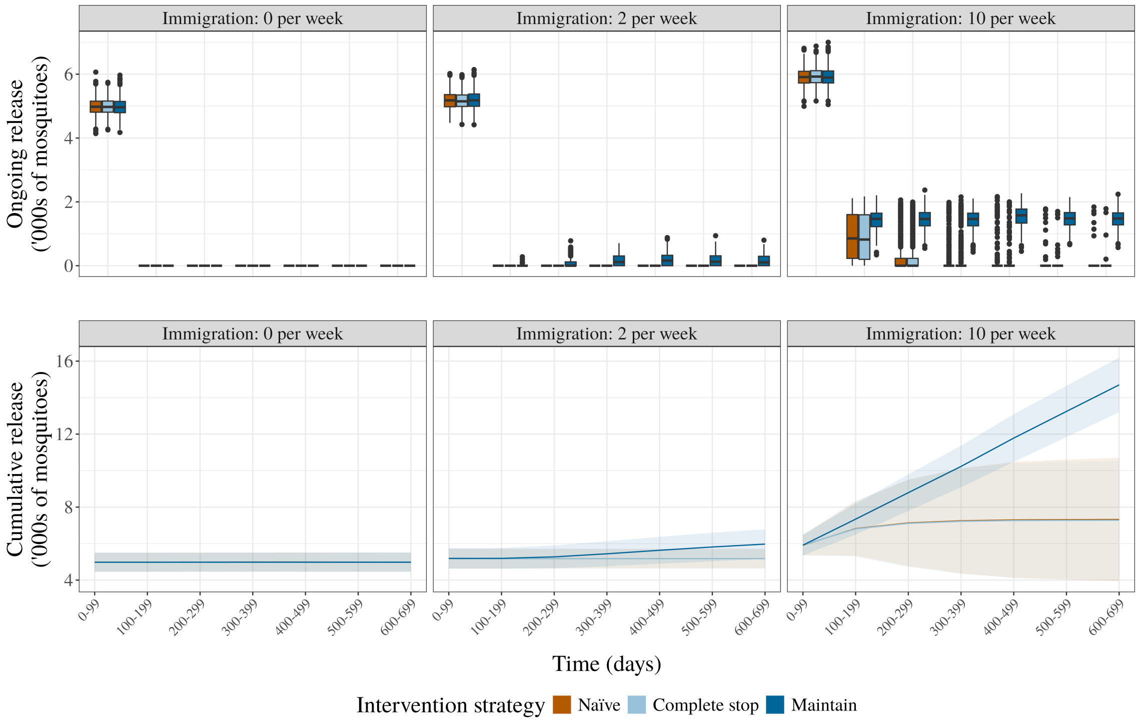

Figure S14: The release in thousands of ARwP mosquitoes as a proxy for cost under different intervention strategies and immigration rates with no age-related decay. The x-axis represents the time in 100-day intervals up until day 700. The top row shows the ongoing cost every hundred days, measured as the number of mosquitoes released every 100 days for each intervention strategy (naïve, complete stop, and maintain). The bottom row illustrates the cumulative release (in thousands of mosquitoes) over the entire period for each strategy. The model parameters are the expected values defined in Table S1.

References

[1] Barry W. Alto and Steven A. Juliano. Precipitation and Temperature Effects on Populations of *Aedes albopictus* (Diptera: Culicidae): Implications for Range Expansion. *Journal of Medical Entomology*, 38(5):646–656, Sep 2001. ISSN 0022-2585. doi: 10.1603/0022-2585-38.5.646. URL https://doi.org/10.1603/0022-2585-38.5.646.

[2] Barry W. Alto and Steven A. Juliano. Temperature Effects on the Dynamics of *Aedes albopictus* (Diptera: Culicidae) Populations in the Laboratory. *Journal of Medical Entomology*, 38(4):548–556, Jul 2001. ISSN 0022-2585. doi: 10.1603/0022-2585-38.4.548. URL https://doi.org/10.1603/0022-2585-38.4.548.

[3] Maurizio Calvitti, Francesca Marini, Angiola Desiderio, Arianna Puggioli, and Riccardo Moretti. *Wolbachia* Density and Cytoplasmic Incompatibility in *Aedes albopictus*: Concerns with Using Artificial *Wolbachia* Infection as a Vector Suppression Tool. *PLOS ONE*, 10(3):e0121813, Mar 2015. ISSN 1932-6203. doi: 10.1371/journal.pone.0121813. URL https://journals.plos.org/plosone/article?id=10.1371/journal.pone.0121813. Publisher: Public Library of Science.

[4] Beniamino Caputo, Riccardo Moretti, Chiara Virgillito, Mattia Manica, Elena Lampazzi, Giulia Lombardi, Paola Serini, Verena Pichler, Nigel W Beebe, Alessandra Della Torre, et al. A bacterium against the tiger: further evidence of the potential of noninundative releases of males with manipulated Wolbachia infection in reducing fertility of *Aedes albopictus* field populations in Italy. *Pest management science*, 79(9):3167–3176, 2023.

[5] Mattia Manica, Federico Filipponi, Antonello D'Alessandro, Alessia Screti, Markus Neteler, Roberto Rosà, Angelo Solimini, Alessandra della Torre, and Beniamino Caputo. Spatial and Temporal Hot Spots of *Aedes albopictus* Abundance inside and outside a South European Metropolitan Area. *PLOS Neglected Tropical Diseases*, 10(6):e0004758, Jun 2016. ISSN 1935-2735. doi: 10.1371/journal.pntd.0004758. URL https://journals.plos.org/plosntds/article?id=10.1371/journal.pntd.0004758. Publisher: Public Library of Science.

[6] H. Nur Aida, A. Abu Hassan, A. T. Nurita, M. R. Che Salmah, and B. Norasmah. Population analysis of *Aedes albopictus* (Skuse) (Diptera:Culicidae) under uncontrolled laboratory conditions. *Tropical Biomedicine*, 25(2):117–125, Aug 2008. ISSN 0127-5720.

[7] D. E. Pagendam, B. J. Trewin, N. Snoad, S. A. Ritchie, A. A. Hoffmann, K. M. Staunton, C. Paton, and N. Beebe. Modelling the *Wolbachia* incompatible insect technique: strategies for effective mosquito population elimination. *BMC Biology*, 18(1):161, Nov 2020. ISSN 1741-7007. doi: 10.1186/s12915-020-00887-0. URL https://doi.org/10.1186/s12915-020-00887-0.

[8] Laura Vavassori, Adam Saddler, and Pie Müller. Active dispersal of *Aedes albopictus*: a mark-release-recapture study using self-marking units. *Parasites & Vectors*, 12(1):583, Dec 2019. ISSN 1756-3305. doi: 10.1186/s13071-019-3837-5. URL https://doi.org/10.1186/s13071-019-3837-5.
